# Requirement of CRAMP for mouse macrophages to eliminate phagocytosed *E. coli* through an autophagy pathway

**DOI:** 10.1242/jcs.252148

**Published:** 2021-03-08

**Authors:** Keqiang Chen, Teizo Yoshimura, Wanghua Gong, Cuimeng Tian, Jiaqiang Huang, Giorgio Trinchieri, Ji Ming Wang

**Affiliations:** 1 Laboratory of Cancer ImmunoMetabolism, Center for Cancer Research, National Cancer Institute at Frederick, Frederick, MD 21702, USA; 2 Department of Pathology and Experimental Medicine, Graduate School of Medicine, Dentistry and Pharmaceutical Sciences, Okayama University, Okayama 700-8558, Japan; 3 Basic Research Program, Leidos Biomedical Research, Inc., Frederick, MD 21702, USA; 4 Beijing Tuberculosis and Thoracic Tumor Research Institute/Beijing Chest Hospital, Capital Medical University, Beijing 101149, China; 5 College of Life Sciences, Beijing Jiaotong University, Beijing 100044, China; 6 Laboratory of Integrative Cancer Immunology, Center for Cancer Research, National Cancer Institute, Bethesda, MD 20892, USA

**Keywords:** CRAMP, Macrophages, *E. coli*, Elimination, Autophagy

## Abstract

Host-derived antimicrobial peptides play an important role in the defense against extracellular bacterial infections. However, the capacity of antimicrobial peptides derived from macrophages as potential antibacterial effectors against intracellular pathogens remains unknown. In this study, we report that normal (wild-type, WT) mouse macrophages increased their expression of cathelin-related antimicrobial peptide (CRAMP, encoded by *Camp*) after infection by viable *E. coli* or stimulation with inactivated *E. coli* and its product lipopolysaccharide (LPS), a process involving activation of NF-κB followed by protease-dependent conversion of CRAMP from an inactive precursor to an active form. The active CRAMP was required by WT macrophages for elimination of phagocytosed *E. coli*, with participation of autophagy-related proteins ATG5, LC3-II and LAMP-1, as well as for aggregation of the bacteria with p62 (also known as SQSTM1). This process was impaired in *CRAMP^−/−^* macrophages, resulting in retention of intracellular bacteria and fragmentation of macrophages. These results indicate that CRAMP is a critical component in autophagy-mediated clearance of intracellular *E. coli* by mouse macrophages.

## Abstract (precis)

**Summary:** CRAMP is a critical component in autophagy-mediated clearance of phagocytosed *E. coli* by mouse macrophages.

## INTRODUCTION

Macrophages comprise an essential part of the innate immune system in response to bacterial infections (Rosenberger and Finlay, 2003). Because macrophages are highly phagocytic and are readily confronted by pathogenic bacteria, they must be equipped with effective mechanisms for either killing bacteria or controlling their replication to avoid becoming a reservoir of infection. For example, colon macrophages residing in the subepithelial lamina propria (LP) represent the first line of defense against invading pathogens, hence acting as crucial sentinels for the maintenance of colon homeostasis (Mowat and Agace, 2014). *E. coli* belongs to the family of *Enterobacteriaceae* in the phylum Proteobacteria*,* which constitutes a minor fraction of the microbiome found in the human gastrointestinal tract (Bailey et al., 2010). However, *E. coli* is the most common cause of intestinal and extra-intestinal diseases (Conway and Cohen, 2015; Foster, 2004; Katouli, 2010). Many host factors, including inflammation and genetic predisposition, markedly alter the colonic microbial composition and support the growth of either resident or introduced aerobic bacteria, particularly those of the *Enterobacteriaceae* family (Lupp et al., 2007). The number of *E. coli* is expanded and the *E. coli* serotypes are increased in inflammatory bowel diseases (IBD) (Bambou et al., 2004; Martin et al., 2004; Rhodes, 2007; Zhang et al., 2017) and in colorectal cancer tissues, which is associated with DNA damage in epithelial cells (Arthur et al., 2012; Dejea et al., 2018). Previous studies have shown that adherent–invasive *E. coli* (AIEC) plays a central role in the pathogenesis of human IBD and colon cancer (Martin et al., 2004; Raisch et al., 2014; Sarabi Asiabar et al., 2018). AIEC bacteria are able to replicate within epithelial cells and macrophages, and defects in autophagy impair the ability of epithelial cells and macrophages to control AIEC replication (Lapaquette et al., 2012). However, the role of cathelin-related antimicrobial peptide (CRAMP, encoded by *Camp*) in macrophage elimination of intracellular *E. coli* remains unknown.

Autophagy is utilized by macrophages to eliminate intracellular or phagocytosed bacteria (Deretic, 2011; Levine et al., 2011), as well as to exert a housekeeping function, and therefore plays a protective role in maintaining cellular homeostasis (Moreau et al., 2010). The autophagy process in macrophages is activated in response to many stress conditions, including starvation, endoplasmic reticulum dysfunction, oxidative damage, and exposure to chemicals, radiation and hypoxia (Mizushima and Komatsu, 2011). Bacterial infection and inflammation are also able to trigger autophagy in macrophages and other immune cells (Saitoh and Akira, 2010). When activated in infected macrophages, autophagy promotes the clearance of pathogenic bacteria including *Salmonella typhimurium*,* Shigella flexneri* (Deretic and Levine, 2009) and *Mycobacterium tuberculosis* (Rekha et al., 2015). Bacteria initiate autophagy in macrophages mainly via their pathogen-associated molecular patterns (PAMPs) and damage-associated molecular patterns (DAMPs). Cell surface recognition and cytosolic sensing of invading pathogens by these molecules result in signaling cascades that promote rapid and localized autophagy machinery assembly. For instance, as a cytosolic sensor in macrophages, cGAS recognizes bacterial DNA to trigger autophagy activation, resulting in ubiquitylation of the bacteria or its phagosome by ubiquitin ligases Parkin and Smurf1. Ubiquitin chains subsequently bind to autophagy adaptors such as p62 (also known as SQSTM1 or A170) and NDP52 (also known as CALCOCO2) that recruit LC3 (MAP1LC3B) to deliver bacteria into an autophagosome. In addition, damaged phagosomes are also targeted by autophagy via the recognition of host glycan present in the phagosomal lumen through cytosolic lectins of the galectin family. The process is tightly regulated by more than 30 autophagy-related gene products (ATGs). Upon autophagy activation, ATGs, serine/threonine kinase ULK1, and Beclin-1, in association with ATG14 and type III phosphatidylinositol 3-kinase VPS34, promote the formation of a cup-shaped isolation membrane to engulf the cargo to form a double-membrane autophagosome, which then fuses with lysosomes to form an autolysosome in which the engulfed cargo is degraded (Klionsky, 2010). However, the role of autophagy in macrophage elimination of phagocytosed *E. coli* is unclear.

LL-37 (also known as CAMP) in human and its mouse ortholog CRAMP are cathelin-related antimicrobial peptides, which belong to a family of host-derived antibacterial polypeptides (Zhang et al., 2019). LL-37 and CRAMP are amphipathic α-helical peptides that bind to negatively charged groups of the bacterial outer membrane causing disruption of the cell wall (Scott and Hancock, 2000). In mouse macrophages, CRAMP is upregulated by infection with intracellular pathogens such as *S. typhimurium* (Rosenberger et al., 2004) or *Mycobacterium* *smegmatis* (Sonawane et al., 2011). CRAMP is an essential component in host anti-microbial defense; it directly impairs the replication of intracellular pathogens, therefore assisting their killing by macrophages (Rosenberger et al., 2004; Sonawane et al., 2011), as well as participating in the autophagy process to eliminate bacteria. In human macrophages, LL-37 is not only directly bactericidal but also serves as a mediator of vitamin D3-induced autophagy to activate the transcription of autophagy-related genes *BECN1* (encoding Beclin-1) and *ATG5*, therefore indirectly participating in the elimination of intracellular bacteria (Yuk et al., 2009). However, it is not known whether CRAMP in mouse macrophages acts as a part of an antibacterial effector mechanism against phagocytosed *E. coli*.

In this study, we investigated the expression of CRAMP in mouse macrophages after stimulation with live or inactivated *E. coli* and its role in the elimination of intracellular inactivated *E. coli* by using cells derived from the bone marrow (BM) of *CRAMP^−/−^* mice. We also explored the relationship between CRAMP and autophagy in mouse macrophages. Our results indicate essential participation of CRAMP in mouse macrophage elimination of intracellular *E. coli* through autophagy processes.

## RESULTS

### Stimulation of CRAMP production in macrophages by *E. coli* products

**Fig. 1.**
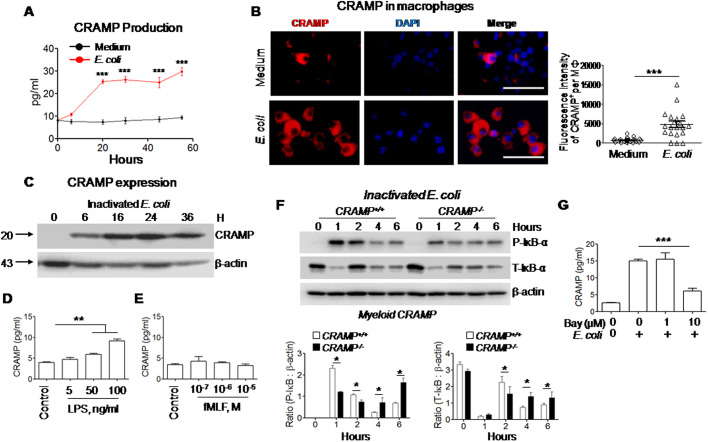
**CRAMP production induced by *E. coli* products in macrophages.** (A) Production of CRAMP by macrophages. Macrophages from BM of myeloid *CRAMP^+/+^* control mice were seeded in 96-well plates at 1.5×10^5^/well and infected with *E. coli* O22H8 (MOI=80) or incubated in medium. The supernatants were harvested at 0, 6, 20, 30, 45 and 55 h for measurement of CRAMP using ELISA. *n*=3 per group. ***P<0.001, significantly increased CRAMP in supernatants of *E. coli*-infected cells compared to those of cells treated with medium alone at 20, 30, 45 and 55 h (two-way ANOVA test with Bonferroni post-hoc test). (B) Detection of increased CRAMP in macrophages infected by *E. coli* O22H8 (MOI=10) for 4 h. Red, CRAMP; blue, DAPI. Scale bars: 30 µm. Right panel: quantitation of CRAMP-positive staining spots per macrophage (Mφ). The immunofluorescence intensity per macrophage is shown, *n*=20–22 macrophages per group. ***P<0.001 (paired, two-tailed Student's *t*-test). (C) Upregulation of CRAMP in macrophages stimulated with inactivated *E. coli* O22H8. Macrophages from BM of myeloid *CRAMP^+/+^* control mice were stimulated with inactivated *E. coli* O22H8 (MOI=10) at 37°C then were lysed at the indicated time points. The cell lysates were measured for CRAMP by western blotting. β-actin is shown as a loading control. (D,E) LPS- or fMLF-stimulated CRAMP production by macrophages. Macrophages from BM of myeloid *CRAMP^+/+^* control mice were seeded in 96-well plates at 1.5×10^5^/well and stimulated with LPS (D) or fMLF (E) at the indicated concentrations for 24 h. The supernatants were measured for CRAMP using ELISA. *n*=3 per group. **P<0.01 (one-way ANOVA with Kruskal–Wallis test). (F) Reduced IκB activation in macrophages from BM of myeloid *CRAMP^−/−^* mice, compared with that in macrophages from BM of *CRAMP^+/+^* mice, by treatment with inactivated *E. coli* O22H8 (MOI=10) for the indicated times. P-IκB-α, phosphorylated (active) IκB; T-IκB-α, total IκB. β-actin is shown as a loading control. Lower panels: the ratio of P-IκB-α to β-actin (left) and ratio of T-IκB-α to β-actin (right). *P<0.05 (paired, two-tailed Student's *t*-test). (G) IκB inhibitor BAY117082 attenuated CRAMP production by control macrophages. Macrophages were seeded in 96-well plates at 1.5×10^5^/well and cultured in the presence of different concentrations of BAY117082 (Bay) for 1 h at 37°C before stimulation with inactivated *E. coli* O22H8 (MOI=10) for an additional 20 h. The supernatants were harvested for measurement of CRAMP using ELISA. ***P<0.001 (one-way ANOVA with Kruskal–Wallis test). Quantitative data are presented as mean±s.e.m.

To obtain evidence for the importance of CRAMP for macrophages to eliminate phagocytosed *E. coli*, we generated macrophages from BM cells of *CRAMP^+/+^* control mice. After infection with *E. coli* isolated from the feces of naïve mice, the production of CRAMP by control macrophages progressively increased and reached the maximal level by 20 h (Fig. 1A,B). Inactivated *E. coli* also stimulated *CRAMP^+/+^* macrophages to produce CRAMP, as confirmed by western blotting (Fig. 1C).

In addition, lipopolysaccharide (LPS), as the principal component of Gram-negative bacteria such as *E. coli* (Raetz and Whitfield, 2002), dose-dependently stimulated *CRAMP^+/+^* control macrophages to produce CRAMP (Fig. 1D). In contrast, another product of *E. coli*, the chemotactic peptide N-formyl-methionyl-leucyl-phenylalanine (fMLF; Schiffmann et al., 1975), failed to stimulate macrophages to produce CRAMP (Fig. 1E).

We further revealed that stimulation of control macrophages by inactivated *E. coli* induced rapid phosphorylation of IκB-α, shown by an increase in total IκB-α due to *de novo* synthesis (Karin, 1999). Fig. 1F showed that the intensity of phosphorylation of IκB-α (also known as NFKBIA) induced by inactivated *E. coli* at 1 h and 2 h was significantly higher in *CRAMP^+/+^* control macrophages than in *CRAMP^−/−^* macrophages. At 6 h, levels of phospho-IκB-α began to elevate again but there was no significant difference between *CRAMP^+/+^* control and *CRAMP^−/−^* macrophages. Also, the intensity of *de novo* synthesis of total IκB-α was higher at 2 h after stimulation with inactivated *E. coli* in *CRAMP^+/+^* control macrophages than in *CRAMP^−/−^* macrophages. The CRAMP production by *CRAMP^+/+^* control macrophages in response to inactivated *E. coli* was attenuated by a selective IκB-α inhibitor BAY117082 (Fig. 1G). Thus, activation of NF-κB is critical for macrophages to produce CRAMP in response to stimulation by *E. coli* and its product LPS.

### Requirement of CRAMP for macrophages to eliminate phagocytosed *E. coli*

**Fig. 2.**
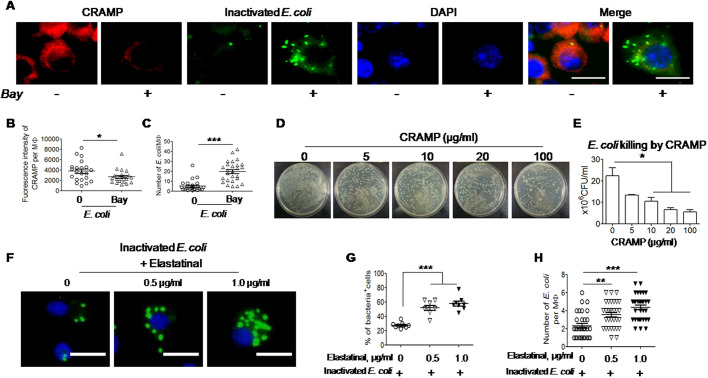
**Requirement of CRAMP for macrophages to eliminate phagocytosed *E. coli.*** (A–C) Reduction in CRAMP production and degradation of phagocytosed inactivated *E. coli* O22H8 in macrophages treated with BAY117082 (Bay). (A) RAW 264.7 cells (mouse macrophage line) were seeded in 35 mm dishes with 14 mm coverslips at 1×10^6^ cells/dish. The cells were then cultured in the presence or absence of 10 μm BAY117082 for 1 h at 37°C before stimulation with inactivated *FITC*-labeled *E. coli* O22H8 (MOI=10) for an additional 20 h. The cells were then stained with an anti-CRAMP antibody. Red, CRAMP; green, inactivated *E. coli*–FITC; blue, DAPI. Scale bars: 10 μm. (B) Reduced CRAMP production in *CRAMP^+/+^* control macrophages treated with BAY117082. Shown is the CRAMP^+^ immunofluorescence intensity per macrophage (Mφ). (C) Delayed elimination of inactivated *E. coli* by macrophages treated with BAY117082. *P<0.05, ***P<0.001 (paired, two-tailed Student's *t*-test). (D) Killing of *E. coli* O22H8 by synthetic CRAMP. *E. coli* O22H8 was diluted to a concentration of 5×10^4^ in 100 μl/well in 96-well plates. Various concentrations of synthetic CRAMP were added to the culture for incubation at 37°C for 2 h. The bacteria cultured with or without CRAMP were serially diluted at 1:5 with sterile PBS and plated on LB agar in triplicates to examine colony formation. (E) Quantitation of the capacity of CRAMP at different concentrations to kill *E. coli* O22H8. *P<0.05 (one-way ANOVA with Kruskal–Wallis test). (F–H) Reduction of degradation of intracellular inactivated *E. coli* O22H8 by macrophages in the presence of an elastase inhibitor, elastatinal. (F) RAW 264.7 cells were seeded in 35 mm dishes with 14 mm coverslips at 1×10^6^ cells/dish, then were cultured in the absence or presence of elastatinal (0.5 or 1 μg/ml) for 1 h at 37°C before stimulation with inactivated *E. coli*–FITC (MOI=10) for an additional 20 h. Green, inactivated *E. coli*–FITC; blue, DAPI. Scale bars: 10 μm. (G) Quantitation of the cells positive with bacteria (%). (H) Quantitation of bacteria number per RAW 264.7 cell. The experiments were repeated three times, *n*=8 fields per group. **P<0.01, ***P<0.001 (one-way ANOVA with Kruskal–Wallis test). Quantitative data are presented as mean±s.e.m.

To examine the role of CRAMP in macrophage elimination of phagocytosed *E. coli*, a mouse RAW 264.7 cell line used as an *in vitro* model was co-cultured with inactivated *E. coli* for 20 h. RAW 264.7 cells expressed a high level of CRAMP and had few endocytosed inactivated *E. coli* (Fig. 2A,B). Preincubation of RAW 264.7 cells with BAY117082 reduced the production of CRAMP and increased the number of phagocytosed inactivated *E. coli* within the cells (Fig. 2A,C). The bactericidal activity of CRAMP was also shown by a synthetic CRAMP peptide, which directly killed *E. coli in vitro* (Fig. 2D,E).

CRAMP is normally stored in lysosomes of macrophages as an inactive precursor, which is converted to an active form through cleavage by proteases (Shinnar et al., 2003; Zanetti, 2004) such as intracellular elastase-like serine protease (Rosenberger et al., 2004). We found that elastatinal, an elastase inhibitor, attenuated the capacity of macrophages to eliminate phagocytosed *E. coli* (Fig. 2F–H). Therefore, CRAMP production and conversion are critical for macrophages to eliminate both phagocytosed and extracellular *E. coli*.

### Reduced capacity of *CRAMP^−/−^* macrophages to eliminate phagocytosed *E. coli*

**Fig. 3.**
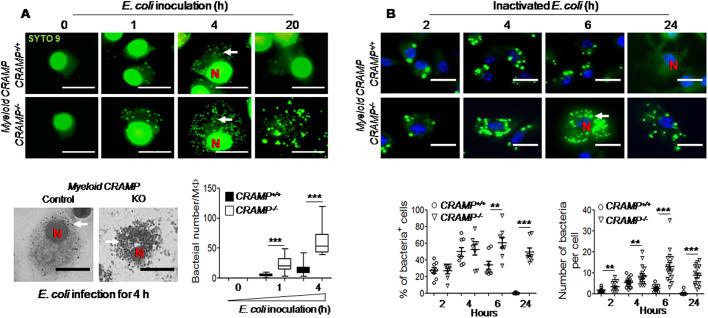
**Attenuation of the capacity of *CRAMP^−/−^* macrophages to eliminate phagocytosed *E. coli.*** (A) Reduced killing of phagocytosed *E coli* O22H8 by *CRAMP^−/−^* macrophages. *CRAMP^+/+^* and *CRAMP^−/−^* macrophages were seeded in 35 mm dishes with 14 mm coverslips at 1×10^6^ cells/dish. The cells were then infected with *E. coli* O22H8 (MOI=5) for 1 h, before treatment with gentamicin (50 μg/ml) for 30 min. The cells were re-cultured and harvested at the indicated time points for staining with SYTO 9 to reveal intracellular bacteria. Top panels: 0 h, macrophages in medium only; 1, 4 and 20 h, macrophages infected with *E. coli*. Lower left panel: inverted grayscale image representing the results of fluorescence shown at 4 h after *E. coli* infection of *CRAMP^+/+^* (control) and *CRAMP^−/−^* (KO) macrophages. N, nuclei; white arrows, *E. coli*. Scale bars: 10 µm. Lower right panel: quantitation of *E. coli* in each macrophage (Mφ). Boxes show the interquartile range with the median indicated. Whiskers show the range. ***P<0.001, significantly higher number of *E. coli* O22H8 in *CRAMP^−/−^* macrophages (one-way ANOVA with Kruskal–Wallis test). (B) Failure of *CRAMP^−/−^* macrophages to eliminate phagocytosed inactivated *E. coli* O22H8*.* Upper panels: *CRAMP^+/+^* and *CRAMP^−/−^* macrophages were seeded in 35 mm dishes with 14 mm coverslips at 1×10^6^ cells/dish. The cells were stimulated with FITC-labeled inactivated *E. coli* O22H8 (MOI=10) for 1 h. The cells were washed and re-incubated with fresh medium and harvested at the indicated time points. Green, inactivated *E. coli* O22H8; blue, DAPI. N, nuclei; white arrows, *E. coli*. Scale bars: 10 μm. Lower left panel: quantitation of macrophages (%) that had phagocytosed inactivated *E. coli* O22H8. Lower right panel: quantitation of phagocytosed inactivated *E. coli* O22H8 in single macrophages. The experiments were repeated three times, *n*=7–12 fields/group. Data are presented as mean±s.e.m. **P<0.01; ***P<0.001 (one-way ANOVA with Kruskal–Wallis test).

*CRAMP^−/−^* macrophages were used to examine the capacity of CRAMP to eliminate intracellular *E. coli.* CRAMP expression was significantly reduced in *CRAMP^−/−^* macrophages as compared to levels in *CRAMP^+/+^* control macrophages (Fig. S1). The number of *E. coli* in *CRAMP^−/−^* macrophages was significantly increased as compared with the number in *CRAMP*^+/+^ macrophages 4 h after infection. By 20 h, many *CRAMP^−/−^* macrophages disintegrated, allowing the formation of numerous extracellular bacterial colonies. By contrast, only a small number of bacteria were visible in macrophages from *CRAMP^+/+^* control macrophages (Fig. 3A). The impaired capacity of *CRAMP^−/−^* macrophages to eliminate intracellular *E. coli* was also supported by the observation that when macrophages infected with *E. coli* were treated with gentamicin to kill extracellular *E. coli* then cultured in the presence of gentamicin for 20 h, *CRAMP^−/−^* macrophages showed a higher number of *E. coli* [colony-forming unit (CFU)/ml] intracellularly than *CRAMP^+/+^* control macrophages (Fig. S2).

In addition, when macrophages were co-cultured with inactivated *E. coli*, the percentage of the cells phagocytosing inactivated *E. coli* and the number of inactivated *E. coli* per cell reached a peak at 4 h, followed by a reduction at 6 h, with only very few bacteria visible at 24 h in *CRAMP^+/+^* control macrophages (Fig. 3B, upper panels). In contrast, in *CRAMP*^−/−^ macrophages, the percent of the cells phagocytosing inactivated *E. coli* and the number of inactivated *E. coli* per cell reached a peak at 6 h, and a considerable number of bacteria remained in the cells at 24 h **(**Fig. 3B, lower panels). These results indicate that CRAMP was required for macrophages to eliminate phagocytosed *E. coli* in a timely manner and that deletion of CRAMP impaired this capacity.

### Involvement of autophagy pathway in CRAMP-mediated elimination of phagocytosed *E. coli* by macrophages

**Fig. 4.**
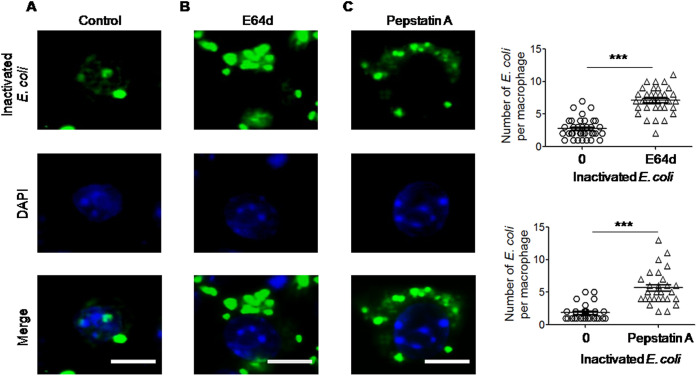
**Delayed elimination of inactivated *E. coli* O22H8 in macrophages by inhibitors of auto-phagolysosomes.** RAW264.7 mouse macrophages were pretreated with E64d (1 μg/ml) or pepstatin A (10 μg/ml) for 1 h at 37°C before stimulation with FITC-labeled inactivated *E. coli* O22H8 (MOI=10) for an additional 20 h. (A) Control cell group. (B) E64d-treatment cell group. (C) Cells treated with Pepstatin. Green, inactivated *E. coli*–FITC; blue, DAPI. Scale bars: 5 μm. Upper right panel: delayed elimination of phagocytosed inactivated *E. coli* by E64d-treated cells. Lower right panel: delayed elimination of inactivated *E. coli* by pepstatin A-treated cells. Data are presented as mean±s.e.m. ***P<0.001 (paired two-tailed Student's *t*-test).

We then tested whether lysosomal hydrolases in macrophages are required for autophagic elimination of inactivated *E. coli*. Treatment of RAW264.7 mouse macrophages with E64d, an inhibitor of cathepsins B and L, or with pepstatin A, an inhibitor of cathepsin D, which suppress autolysosomal digestion, protected *E. coli* from autophagic elimination by the cells (Fig. 4A–C). Thus, lysosomal proteases are important for autophagic degradation of inactivated *E. coli* by macrophages.

**Fig. 5.**
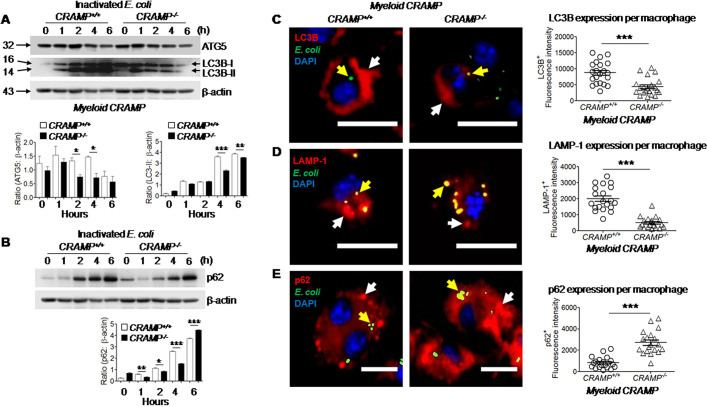
**Involvement of the autophagy pathway in CRAMP-mediated elimination of inactivated *E. coli* by macrophages.** (A) Activation of autophagy-related proteins ATG5 and LC3B-II in macrophages. *CRAMP^+/+^* and *CRAMP^−/−^* macrophages were cultured in the presence of inactivated *E. coli* O22H8 (MOI=10) at 37°C then lysed at the indicated time points. The cell lysates were assayed for ATG5, LC3-I and LC3-II proteins by western blotting. β-actin is shown as a loading control. Size markers are shown in kDa. Lower panels: quantification of ATG5:β-actin ratio (left) and LC3-II:β-actin ratio (right). *P<0.05; **P<0.01; ***P<0.001 (paired, two-tailed Student's *t*-test). (B) Activation of autophagy-related protein p62 in macrophages, assayed as described for A. Lower panel: quantification of p62:β-actin ratio. *P<0.05; **P<0.01; ***P<0.001 (paired, two-tailed Student's *t*-test). (C–E) *CRAMP^+/+^* and *CRAMP^−/−^* macrophages were seeded in 35 mm dishes with 14 mm coverslips at 1×10^6^ cells/dish. The cells were stimulated with *FITC*-labeled inactivated *E. coli* O22H8 (MOI=10) for 12 h. The samples were fixed with 4% neutral formalin for 5 min, stained with primary antibodies (1:100, anti-LC3B, anti-LAMP-1 and anti-p62 antibodies) followed by a biotinylated secondary antibody and streptavidin–PE. DAPI was used to stain nuclei. (C) Reduced levels of LC3B protein in *CRAMP^−/−^* macrophages after stimulation with inactivated *E. coli* O22H8. Red, LC3B; green, *E. coli*; blue, DAPI. White arrows, LC3B; yellow arrows, *E. coli*. Right panel: quantitation of LC3B^+^ fluorescence intensity per macrophage. (D) Reduced levels of LAMP-1 protein in *CRAMP^−/−^* macrophages after stimulation with inactivated *E. coli* O22H8. Red, LAMP-1; green, *E. coli*; blue, DAPI. White arrows, LAMP-1; yellow arrows, *E. coli*. Right panel: quantitation of LAMP-1^+^ fluorescence intensity per macrophage. (E) Increased levels of p62 protein in *CRAMP^−/−^* macrophages after stimulation with inactivated *E. coli* O22H8. Red, p62; green, *E. coli*; blue, DAPI. White arrows, p62; yellow arrows, *E. coli*. Right panel: quantitation of p62^+^ fluorescence intensity per macrophage. ***P<0.001 (paired, two-tailed Student's *t*-test). Quantitative data are presented as mean±s.e.m. Scale bars: 30 μm.

We further found that there was a reduced expression of the autophagy-related protein ATG5, which is involved in the extension of the phagophoric membrane in autophagic vesicles (Matsushita et al., 2007), in *CRAMP*^−/−^ macrophages as compared to expression in *CRAMP^+/+^* control macrophages at 2 and 4 h after incubation with inactivated *E. coli* (Fig. 5A). Under normal conditions, ATG5 forms complexes with ATG12 and ATG16L1, necessary for the conjugation of LC3-I (microtubule-associated proteins 1A/1B light chain 3B, also referred to as LC3B) to phosphatidylethanolamine (PE) to form LC3-II (Otomo et al., 2013). However, LC3-II formation was reduced in *CRAMP^−/−^* macrophages after phagocytosis of inactivated *E. coli* at 4 and 6 h (Fig. 5A). The adaptor protein p62 is an autophagy-targeting molecule that recognizes ubiquitylated cytoplasmic components and delivers them for degradation (Ponpuak et al., 2010). *CRAMP^−/−^* macrophages showed reduced expression of p62 at 1, 2 and 4 h as compared to p62 expression in *CRAMP^+/+^* control macrophages after incubation with inactivated *E. coli* (Fig. 5B). In contrast, intracellular p62 accumulation was higher in *CRAMP^−/−^* macrophages than in *CRAMP^+/+^* control macrophages at 6 h (Fig. 5B), as well as at 8, 20 and 28 h (Fig. S3) after incubation with inactivated *E. coli*, indicating that the production and degradation of p62 induced by *E. coli* was impaired in *CRAMP^−/−^* macrophages.

Participation of CRAMP in the autophagy pathway in macrophages for elimination of inactivated *E. coli* was further demonstrated by reduced fluorescence intensity of LC3B^+^ and LAMP-1^+^ staining and increased fluorescence intensity of p62^+^ staining in *CRAMP^−/−^* macrophages as compared to levels in *CRAMP^+/+^* control macrophages after culture with inactivated *E. coli* for 12 h (Fig. 5C–E). There was a reduced bacterial colocalization with LAMP-1 (Fig. 5D), but increased colocalization between bacteria and p62 (Fig. 5E) in *CRAMP^−/−^* macrophages, indicating that CRAMP deficiency impaired degradation of bacteria conjugated with p62, resulting in retention of intracellular *E. coli*.

## DISCUSSION

**Fig. 6.**
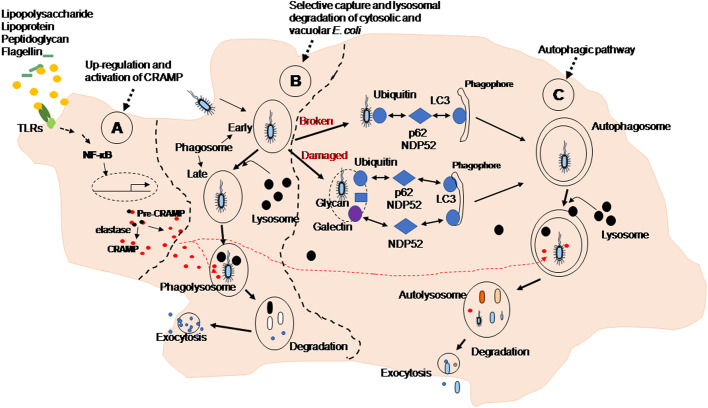
**CRAMP-dependent autophagy to eliminate phagocytosed *E. coli* by macrophages.** (A) Upregulation and activation of CRAMP. Soluble elements from *E. coli* stimulate TLR-mediated signals to activate NF-κB, resulting in upregulation of CRAMP expression in macrophages. The pre-CRAMP is cleaved by elastase to form activated CRAMP. (B) Selective capture and lysosomal degradation of cytosolic and vacuolar *E. coli*. *E. coli* phagocytosed by macrophages are incorporated into phagosomes, fused with lysosomes and degraded. (C) Autophagic pathway. Naked *E. coli* released from phagosomes or damaged phagosomes in macrophages are captured by autophagosomes via ubiquitylation. Autophagosomes fused with lysosomes in the form of autolysosomes are eventually degraded. NDP52: nuclear dot protein 52 kDa. p62: adaptor molecule p62 (also known as A170 or SQSTM1).

In this study, we elucidated previously uncharacterized macrophage effector mechanisms for elimination of phagocytosed *E. coli*. Viable *E. coli* infection and inactivated *E. coli* incubation of mouse macrophages increased intracellular production and extracellular release of CRAMP by activation of NF-κB to trigger autophagy-dependent degradation of the bacteria (as summarized in Fig. 6). Interestingly, although both LPS and the chemotactic peptide fMLF are also products of *E. coli*, only LPS was able to upregulate CRAMP expression in macrophages, indicating that the TLR4 pathway promotes CRAMP expression and secretion, similar to the findings of a previous report using mouse BM-derived mast cells (Li et al., 2009). In addition to LPS stimulation of the TLR4 pathway, phagocytosed *E. coli* release DNA, which induces CRAMP production through interaction with TLR9 via the activation of the signal-regulated kinase (ERK) pathway (Koon et al., 2011).

*E. coli* strain O22H8 from the feces of mice was identified by whole genome sequencing in our study (data not shown). The O22H8 strain was found in the feces of mice under a variety of conditions, such as naïve and dextran sulfate sodium (DSS)-treated mice, was verified as being commensal based on our own results (unpublished data). It has been reported that three substrains of *E. coli* O22H8, isolated from normal healthy cattle, carry *stx1* and *stx2d* genes and are rarely associated with human illness but, in contrast, inhibit expansion of the pathogenic *E. coli* O157H7 strain in humans by adhering to the colon mucosa to cause bloody diarrhea (Martorelli et al., 2017). Thus, commensal *E. coli* is beneficial to both human and animal hosts. However, *E. coli* O22H8 in laboratory mice has rarely been reported previously. *E. coli* belongs to the family *Enterobacteriaceae* of the phylum Proteobacteria, which although constituting a minor fraction of the microbiome found in human gastrointestinal tract (Bailey et al., 2010), is the most common cause of intestinal and extra-intestinal diseases (Conway and Cohen, 2015; Foster, 2004; Katouli, 2010). Many host factors, including inflammation and genetic predisposition, alter the colonic microbial composition and support the growth of either resident or introduced aerobic bacteria, particularly of the *Enterobacteriaceae* family (Lupp et al., 2007) such as *E. coli*, levels of which are elevated in IBD (Bambou et al., 2004; Martin et al., 2004; Rhodes, 2007; Zhang et al., 2017) as well as in colitis-related cancer (CRC) tissues (Arthur et al., 2012; Dejea et al., 2018). Therefore, investigation of the role of CRAMP, as well as its human ortholog LL-37, in elimination of *E. coli* by macrophages has important clinical therapeutic significance.

LL-37 in humans and CRAMP in mouse are expressed by various cells and tissues, such as BM-derived myeloid cells (neutrophils, macrophages) and epithelial cells (Zhang et al., 2019). LL-37 is stored in an intact form in specific granules and contains both a conserved N-terminal cathelin-like region and a highly variable C-terminal region with bactericidal activity (Cowland et al., 1995). The release of active LL-37 from its precursor is mediated by proteinase 3 (Sorensen et al., 2001) or elastase (Gudmundsson et al., 1996). During autophagy, LL-37 synthesized and activated intracellularly is recruited to the autophagosomes (Yuk et al., 2009). The cathelin-like segment of antibacterial cationic proteins appears to be essential for subcellular trafficking through the synthesis apparatuses (ER, Golgi and trans-Golgi network) (Liu and Ganz, 1995). We showed that *E. coli* infection of macrophages increased CRAMP production and that elastatinal blocks the capacity of macrophages to eliminate phagocytosed *E. coli*, suggesting that a critical concentration of active CRAMP is important for macrophage killing of intracellular *E. coli*.

It has been reported that LL-37 plays an important role in intracellular bacterial killing by macrophages. Phenylbutyrate induces LL-37-dependent autophagy and intracellular killing of *M. tuberculosis* in human macrophages (Rekha et al., 2015). Moreover, RNAi-generated mouse *CRAMP^−/−^* macrophages and the cells derived from *CRAMP^−/−^* mouse BM are significantly impaired in their ability to kill mycobacteria (Sonawane et al., 2011). Another intracellular pathogen, *S. typhimurium*, is also inhibited by mouse macrophages via a process dependent on intracellular elastase-like serine protease activity to proteolytically activate CRAMP (Rosenberger et al., 2004). Our study reveals that CRAMP is required for mouse macrophages to kill and eliminate intracellular *E. coli*; a finding supported by our observations that elastatinal, an elastase inhibitor, attenuated the capacity of macrophages to eliminate phagocytosed *E. coli*. *CRAMP^−/−^* macrophages showed reduced expression of autophagy-related proteins ATG5, LC3-II, LAMP-1 and p62 after phagocytosis of *E. coli*. These results further support the role of CRAMP-dependent autophagy in the elimination of phagocytosed *E. coli* by macrophages. Clinical data shows that ileal lesions in Crohn's disease (CD) patients are abnormally colonized by pathogenic AIEC (Lapaquette et al., 2012). AIEC infection of macrophages mobilizes autophagy machinery in the location of phagocytosis to limit intracellular AIEC replication. Impaired ATG16L1, IRGM or NOD2 expression in macrophages increases intracellular AIEC with enhanced secretion of IL-6 and TNF in response to infection. In contrast, forced induction of autophagy decreases the numbers of intra-macrophage AIEC and pro-inflammatory cytokine release (Lapaquette et al., 2012). These results indicate that the autophagy of macrophages is linked to the pathogenesis of IBD.

Our current study showed that CRAMP deficiency was associated with reduced expression of autophagy-related proteins ATG5, LC3-II, and LAMP-1 in macrophages after phagocytosis of *E. coli*. However, the changes in p62 levels were different. p62 is an accessory autophagy-targeting molecule with an unknown role in autophagy. Reported functions for p62 include (Ichimura et al., 2008): (1) Involvement in inclusion body formation when macrophages phagocytose bacteria. (2) Interaction with LC3, which regulates autophagosome formation. p62 delivers specific cytosolic components, including ribosomal protein S30 (rpS30) and additional ubiquitylated proteins, to autophagic organelles and interacts with LC3 through a 11-amino-acid sequence that is rich in acidic and hydrophobic residues, named LC3-recognition sequence (LRS). (3) Formation of the LC3–p62 complex, which is eventually degraded in autolysosomes. In the absence of p62, the cells are unable to generate neo-antibacterial factors, resulting in non-functional autophagy despite maturation, thereby failing to effectively eliminate intracellular bacteria (Ponpuak et al., 2010). The degradation of p62 is a widely used parameter to monitor autophagic activity because p62 binds to LC3 and is selectively degraded during autophagy (Bjorkoy et al., 2005; Pankiv et al., 2007). In our study, CRAMP deficiency reduced the expression of p62 by mouse macrophages when inactivated *E. coli* was phagocytized. After 6 h (at 6, 8, 20, and 28 h), levels of p62 were significantly increased, indicating that the inactivated *E. coli* included in p62 complex were accumulated with delayed degradation in autolysosomes. These data suggest that the autophagic process in macrophages to eliminate intracellular bacteria was impaired in the absence of CRAMP.

Cytokines are signaling molecules as important as hormones and neurotransmitters. When macrophages are exposed to inflammatory stimuli, they secrete cytokines such as TNF, IL-1, IL-6, IL-8 (also known as CXCL8) and IL-12 (Arango Duque and Descoteaux, 2014). In the gut, macrophages residing in the mucosa are able to prevent the entry and colonization of pathogens in the mucosal layer (Weiss and Schaible, 2015). In inflamed gut, inflammatory macrophages are sequentially recruited to mount appropriate immune responses and produce pro-inflammatory cytokines (Na et al., 2019). However, macrophages with autophagy deficiency increased not only the survival of intracellular bacteria, but also the secretion of pro-inflammatory cytokines. The gut lesions in CD patients are abnormally colonized by pathogenic AIEC. In infected macrophages, AIEC induce the recruitment of autophagy machinery components at the site of phagocytosis, and normal autophagy function limits intracellular AIEC replication. Impaired ATG16L1, IRGM or NOD2 expression induces an increase in intracellular AIEC and secretion of IL-6 and TNF in response to AIEC infection. In contrast, forced induction of autophagy decreases the numbers of intra-macrophagic AIEC and pro-inflammatory cytokine release, even in a NOD2-deficient context (Lapaquette et al., 2012). It has also been shown that defects in macrophage-mediated AIEC clearance and increased production of pro-inflammatory cytokines (IL-1β and TNF) in CD patients are linked to polymorphisms related to autophagy such as those in IRGM and ULK-1 (Buisson et al., 2019). *In vivo*, *CRAMP^−/−^* mice show increased susceptibility to *Pseudomonas aeruginosa* (PA) keratitis and enhanced secretion of pro-inflammatory cytokines, including IL-1β, IL-6 and TNF, in PA-infected corneas (Huang et al., 2007). Our present study showed that active CRAMP was required for macrophages to eliminate phagocytosed *E. coli*, with participation of autophagy-related proteins ATG5, LC3-II, and LAMP-1, as well as conjugation of the bacteria with p62. In addition, myeloid *CRAMP^−/−^* mice, but not epithelial *CRAMP^−/−^* mice, show increased plasma levels of IL-1β and IL-6 after DSS intake for 5 d (Chen et al., 2021). We thus hypothesize that stimulating autophagy machinery in macrophages in IBD patients may constitute a plausible therapeutic strategy to concomitantly restrain intracellular bacterial replication and dampen inflammatory responses.

In this study, we have disclosed a link between CRAMP and autophagy in macrophages that assists in the eradication of phagocytosed *E. coli*. These findings shed new light on the potential for development of autophagy-related therapies whereby innate immune responses are mobilized against infection and other diseases (Levine and Kroemer, 2008), including IBD (Haq et al., 2019; Kim et al., 2019; Larabi et al., 2020) and neurodegenerative disorders (Nixon, 2013), that have pathogenetic processes associated with defective autophagy activation.

## MATERIALS AND METHODS

### Mice

Myeloid cell-specific *CRAMP^−/−^* (*LysMCre^+^CRAMP^F/F^*) mice were generated as described previously (Chen et al., 2013b; Yoshimura et al., 2018). Mice used in the experiments were 8–12 weeks old and were allowed free access to standard laboratory chow and tap water. All animals were housed in an air-conditioned room with controlled temperature (22±1°C), humidity (65–70%), and day/night cycle (12 h light, 12 h dark). All animal procedures were governed by the US NIH Guide for the Care and Use of Laboratory Animals (Council, 2011) and were approved by the Animal Care and Use Committee of the NCI-Frederick, National Institutes of Health.

### Generation of *CRAMP*^+/+^ control and *CRAMP*^−/−^ macrophages

BM was flushed from the femurs of euthanized mice with phosphate-buffered saline (PBS) as described previously (Chen et al., 2013a). Red cells were lysed with ACK lysing buffer (Cambrex Bio Science, MD). The cell suspension was centrifuged for 10 min at 1200 rpm (290 ***g***) for 10 min, and the pellet was gently resuspended in Dulbecco's modified essential medium (DMEM; Gibco Invitrogen) supplemented with 2 mM L-glutamine (Gibco. CA), 10 mM HEPES (Gibco, CA), 1 mM sodium pyruvate (Gibco Invitrogen), 10% heat-inactivated FBS (Gibco Invitrogen) and 50 ng/ml M-CSF (Thermo Fisher Scientific, MA). To remove fibroblasts, the cells were cultured in tissue culture dishes (Corning Inc. NY) at 37°C and 5% CO2 overnight. The non–adherent cells were collected, centrifuged and re-cultured in tissue culture dishes (1×10^6^ cells/ml) with addition of DMEM with 50 ng/ml M-CSF for 3 d. The medium was replaced on day 7, and fully differentiated macrophages were harvested*. CRAMP^+/+^* control macrophages were generated from BM of control (*LysMCre^−^CRAMP^F/F^*) mice (referred to as control cells) and *CRAMP^−/−^* macrophages were generated from BM of myeloid cell-specific *CRAMP*^−/−^ (*LysMCre^+^CRAMP^F/F^*) mice. Details of inhibitors and antibodies used are presented in Table S1. LPS (Sigma, MO) and fMLF (Sigma, MO) treatment was carried out as follows. Macrophages from BM of *Myeloid CRAMP*^+/+^ mice were seeded in 96-well plates at 1.5×10^5^/well and stimulated with LPS (0. 5, 50 or 100 ng/ml) or fMLF (0, 10^−7^, 10^−6^ and 10^−5^ M) for 24 h. The supernatants were then measured for CRAMP by ELISA.

### Preparation of *E. coli* O22H8

The colony of *E. coli* O22H8 grown in Violet Red Bile Lactose agar (EMD Millipore, MA) was selected and grown in Luria-Bertani (LB) broth, aerobically at 37°C. *E. coli* was incubated overnight with continuous shaking (200 rpm) in a shaker incubator. The *E. coli* identified was cultured in LB broth at 37°C, 180 rpm for 24 h, then determined for concentration [based on an OD_600 nm_ of 0.4 corresponding to ∼2×10^8^ colony forming units (CFU)/ml; von Köckritz-Blickwede et al., 2010]. *E. coli* suspension was aliquoted in 1 ml volumes and stored at −80°C for future use. For inactivation of *E. coli* O22H8, the bacterial suspension was diluted to 2×10^6^ CFU/ml and 0.4% formalin (by volume ratio) was added and incubated overnight with continuous shaking (200 rpm) at 37°C. A small amount of inactivated *E. coli* (5 μl for each sample) was cultured in Violet Red Bile Lactose agar dishes in an incubator at 37°C overnight, with no *E. coli* growth observed (Landman and van Eck, 2017). The inactivated *E. coli* O22H8 was washed with sterile PBS, and resuspended in sterile PBS at OD_600nm_=0.4, corresponding to ∼2×10^8^ colony forming unit (CFU)/ml), then stored at −80°C for future use. When necessary, live or inactivated *E. coli* O22H8 was labeled with FITC (Isomer I, Sigma) following the manufacturer’s recommended procedures.

### Detection of CRAMP produced by *CRAMP*^+/+^ control macrophages

BM-derived *CRAMP^+/+^* control macrophages were seeded at 1.5×10^5^ cells/well in 96-well plates. Live or inactivated *E. coli* was added into the wells [multiplicity of infection (MOI)=80]. After culture at 37°C in 5% CO_2_, cell supernatant was harvested at indicated time points to measure CRAMP concentration by ELISA using a Mouse CRAMP ELISA Kit (MyBioSource, CA).

### *In vitro* killing of *E. coli* by CRAMP

*E. coli* was diluted at 5×10^4^ cells in 100 µl/well on 96-well plates followed by culture with various concentrations (0.01–100 µg/ml) of synthetic murine CRAMP (Hycult Biotech, PA) at 37°C for 2 h. The bacterial suspension was then serially diluted with PBS and plated on nutrient (LB) agar plates at 37°C for 24 h. The number of *E. coli* treated with CRAMP was quantitated and expressed as the percentage of the number of untreated bacteria as a control.

### Fluorescence detection of macrophage killing of intracellular *E. coli*

BM-derived *CRAMP^+/+^* control and *CRAMP^−/−^* macrophages seeded in 35 mm dishes with 14 mm coverslips in the bottom (MatTek Corporation, MA) were infected with *E. coli* O22H8 at a multiplicity of infection of 5 bacteria per cell (MOI=5) at 37°C in DMEM supplemented with 10% FCS in the presence of M-CSF (50 ng/ml) without antibiotics for 1 h. Then, the cells were treated with gentamicin (50 μg/ml) for 30 min and washed. The cells were re-cultured and fixed at indicated time points followed by staining with SYTO 9 (ThermoFisher, MA).

### LB agar incubation to detect macrophage killing of intracellular *E. coli*

The ability of macrophages to kill phagocytosed *E. coli* was measured by assessing cell-associated *E. coli* after a brief phagocytosis period, then determining how many organisms remain following a longer incubation. The method was as described previously (Drevets et al., 2015), with some modifications. In brief, 2.5×10^6^ *CRAMP^+/+^* or *CRAMP^−/−^* macrophages in 100 μl DMEM, 2.5×10^8^ *E. coli* in 100 μl DMEM (MOI=100) and 50 μl ice-cold normal mouse serum were placed in a snap-cap polypropylene tube, and DMEM was added to give a final volume of 1 ml. The tubes were placed in a shaker at 80 rpm at 37°C for 1 h. Then, the cells were treated with gentamicin (50 μg/ml) for 1 h, then washed and resuspended in 1 ml DMEM with 10% serum in the presence of 5% gentamicin and 50 ng/ml M-CSF, before being incubated at 37°C, 5% CO_2_ for an additional 20 h. Using 0.1 ml of the cell mixture, five 1/10 serial dilutions were made and mixed by vortexing. For each dilution, 0.1 ml, in triplicate, was placed on LB agar (Gibco, MA), and the plates were then inverted and incubated at 37°C for 24–48 h to examine colony formation.

### Elimination of phagocytosed inactivated *E. coli* by *macrophages*

BM-derived *CRAMP^+/+^* and *CRAMP^−/−^* macrophages were seeded in 35 mm dishes with 14 mm coverslips in the bottom at 1×10^6^ cells/dish and co-cultured with FITC-labeled inactivated *E. coli* at a multiplicity of 10 bacteria per cell (MOI=10) at 37°C in DMEM supplemented with 10% FCS in the presence of M-CSF (50 μg/ml). The cells were fixed at 0, 4, 6 and 24 h, or at the indicated time points, then stained with DAPI to label nuclei. The percentage (%) of macrophages containing phagocytosed inactivated *E. coli* and the number of phagocytosed inactivated *E. coli* in a single macrophage at the indicated time points were measured.

### Immunofluorescence

BM-derived *CRAMP^+/+^* control and *CRAMP^−/−^* macrophages were seeded in 35 mm dishes with 14 mm coverslips in the bottom at 1×10^6^ cells/dish and co-cultured with FITC-labeled inactivated *E. coli* at MOI=10 at 37°C in DMEM supplemented with 10% FCS in the presence of M-CSF (50 μg/ml) for 12 h. The cells were fixed with 4% neutrally buffered formalin for 5 min and stained with primary antibodies that specifically recognize mouse LC3B, LAMP-1 and p62 proteins, but not the human or other mammalian forms (1:100; anti-mouse LC3B, LAMP-1 and p62 antibodies; all from Abcam, MA) followed by a biotinylated anti-Ig secondary antibody (BD Biosciences, CA) and streptavidin–PE (Biolegend, CA). DAPI was used to stain nuclei. A total of 4–8 viewing fields from each slide were captured under fluorescence microscopy with an Olympus DP camera and a CellSens (Ver. 1.17) imaging software.

### Western immunoblotting

BM-derived *CRAMP^+/+^* control and *CRAMP^−/−^* macrophages or RAW 264.7 cells (ATCC, VA) grown in 60-mm dishes to sub-confluency were cultured for 3 h in FCS-free MDEM. After treatment with inactivated *E. coli*, the cells were lysed with 1× SDS sample buffer [62.5 mM Tris–HCl (pH 6.8), 2% SDS, 10% glycerol and 50 mM dithiothreitol], then sonicated for 15 s and heated at 100°C for 5 min. Cell lysate was centrifuged at 12,000 rpm (13,523 ***g***) (4°C) for 5 min, and protein concentrations of the supernatants were measured by DC Protein Assay (Bio-Rad). The lysates with titrated proteins were electrophoresed on 10% SDS–PAGE precast gels (Invitrogen, CA) then transferred onto ImmunoBlot polyvinylidene membranes (Bio-Rad), which were blocked with 5% nonfat milk. Phosphorylated IκB-α was detected using phosphospecific antibodies, according to the manufacturer's instructions. After incubation of the membranes with a horseradish peroxidase-conjugated secondary antibody, protein bands were detected with Super Signal Chemiluminescent substrate (Pierce), and the images were quantitated using a G-BOX GeneSnap system (SYNGENE). For detection of total IκB-α, β-actin, ATG5, LC3B, p62 and CRAMP, the membranes were stripped with Restore western blot stripping buffer (Pierce) followed by incubation with specific antibodies (Abcam, MA). Primary antibodies were used at a dilution of 1:1000 for p-IκB-α, IκB-α and β-actin, and 1:100 dilution for ATG5, LC3B, p62 and CRAMP.

### Statistics

All experiments were performed at least three times with triplicate samples. Statistical analysis was performed using GraphPad Prism by two-tailed Student's *t*-test or one-way ANOVA with Kruskal–Wallis Test. Data with error bars represent mean±s.e.m., and *P* values less than 0.05 (*P*<0.05) were considered statistically significant.

## Supplementary Material

Supplementary information

Reviewer comments
