# Supplementary material for: Requirement of CRAMP for mouse macrophages to eliminate phagocytosed E. coli through an autophagy pathway
Source: J Cell Sci. 2021 Mar 8;134(5):jcs252148. doi: 10.1242/jcs.252148 (PMC7970306; doi:10.1242/jcs.252148)
Supplement: Supplementary information [file joces-134-252148-s1.pdf]

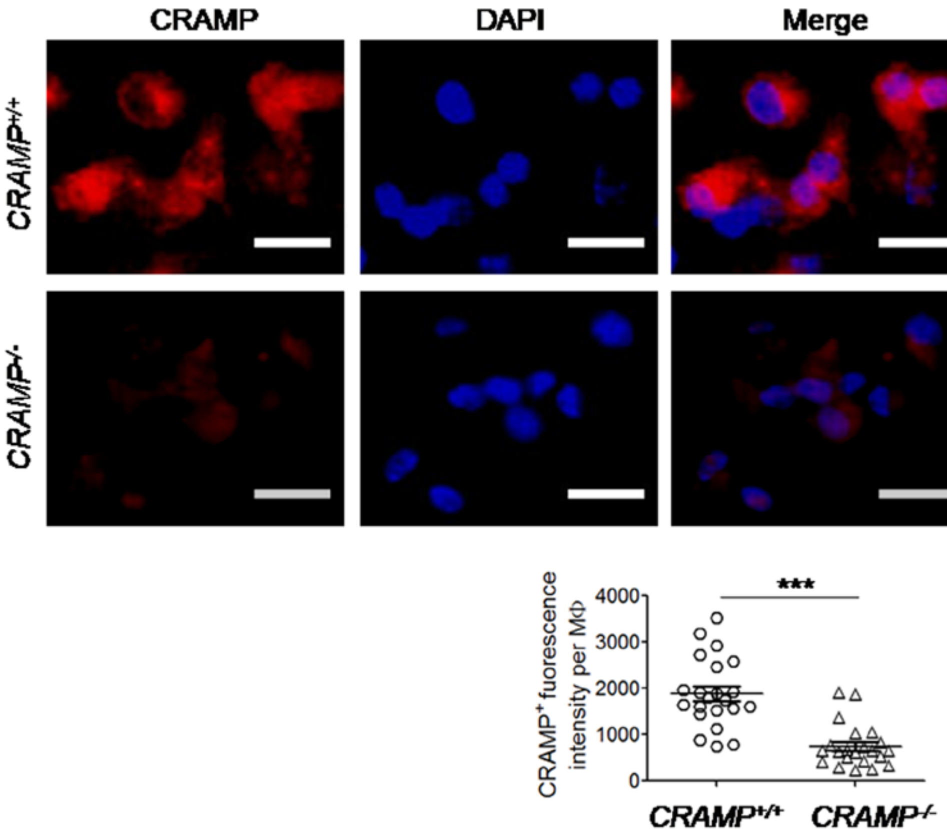

Fig. S1. Reduced expression of CRAMP in macrophages derived from *Myeloid CRAMP*$^{-/-}$ mice.

CRAMP$^{+/+}$ and *CRAMP*$^{-/-}$ macrophages were seeded in 35 mm dishes at 1× 10^6/dish with 14 mm coverslips. The cells were fixed with 4% neutrally buffered formalin for 5 min and stained with an anti-mouse CRAMP antibody followed by a biotinylated anti-Ig secondary antibody (BD Biosciences) and streptavidin-PE. DAPI was used to stain nuclei. **Red:** CRAMP, **blue:** DAPI. Scale bar = 10 µm. **Lower panel**: Quantitation of CRAMP positive staining spots per macrophage. Shown is the immunofluorescence intensity per macrophage, n = 20 macrophages/group. ***P < 0.001.

Journal of Cell Science • Supplementary information

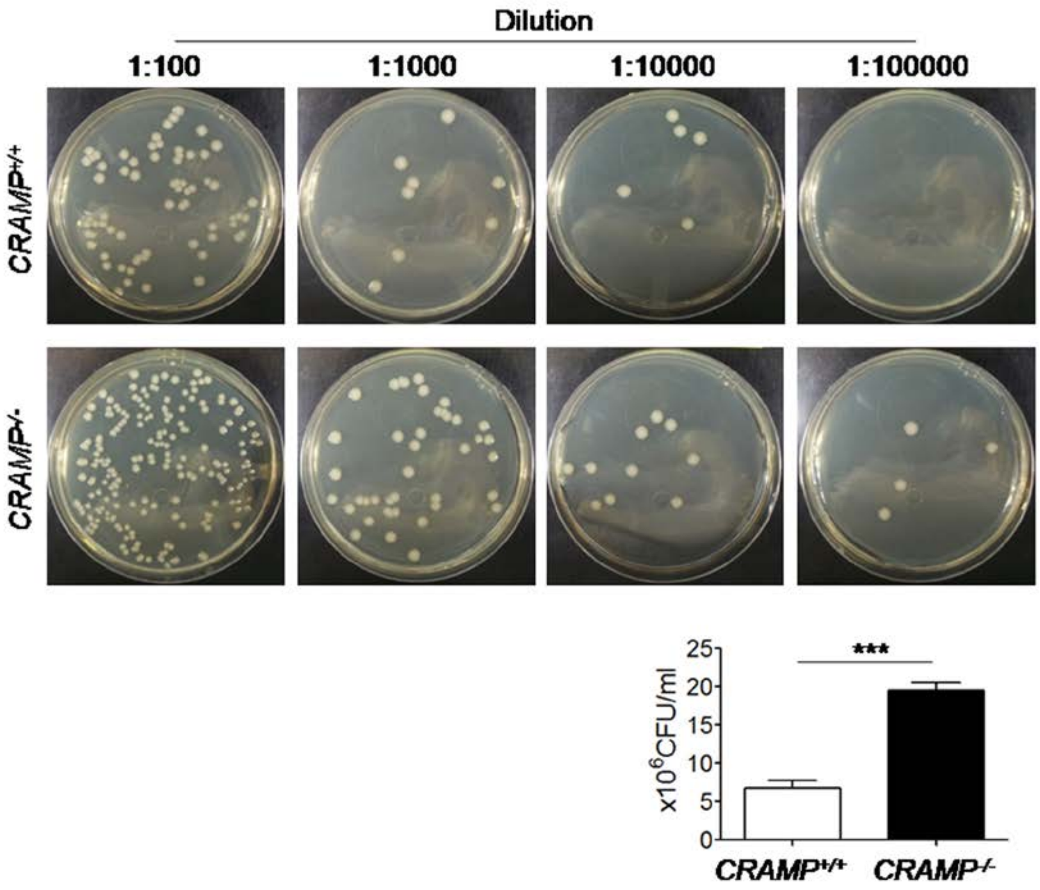

Fig. S2. Attenuated killing of intracellular *E. coli* in *CRAMP*$^{-/-}$ macrophages. *CRAMP*$^{+/+}$ or *CRAMP*$^{-/-}$ macrophages were co-cultured with *E. coli* at MOI= 100 in the snap-cap polypropylene tubes in a shaker at 80 RPM, 37 °C for 1 h. The cells were then treated with gentamicin (50 µg/ml) for 1 h, washed and resuspended in DMEM with 10% FCS in the presence of 5% gentamicin and 50 ng/ml M-CSF followed by further incubation at 37°C, 5% CO_2 for 20 h. Five 1/10 serial dilutions were made in sterile water, which were incubated in triplicates on LB agar at 37°C for 24 to 48 h. **Upper panel:** Representative photos showing the colonies of *E. coli* O22H8 at different dilutions. **Lower panel:** Quantitation of the colonies formed by *E. coli* O22H8 at 1:100 dilution on LB agar. ***P < 0.001.

Journal of Cell Science • Supplementary information

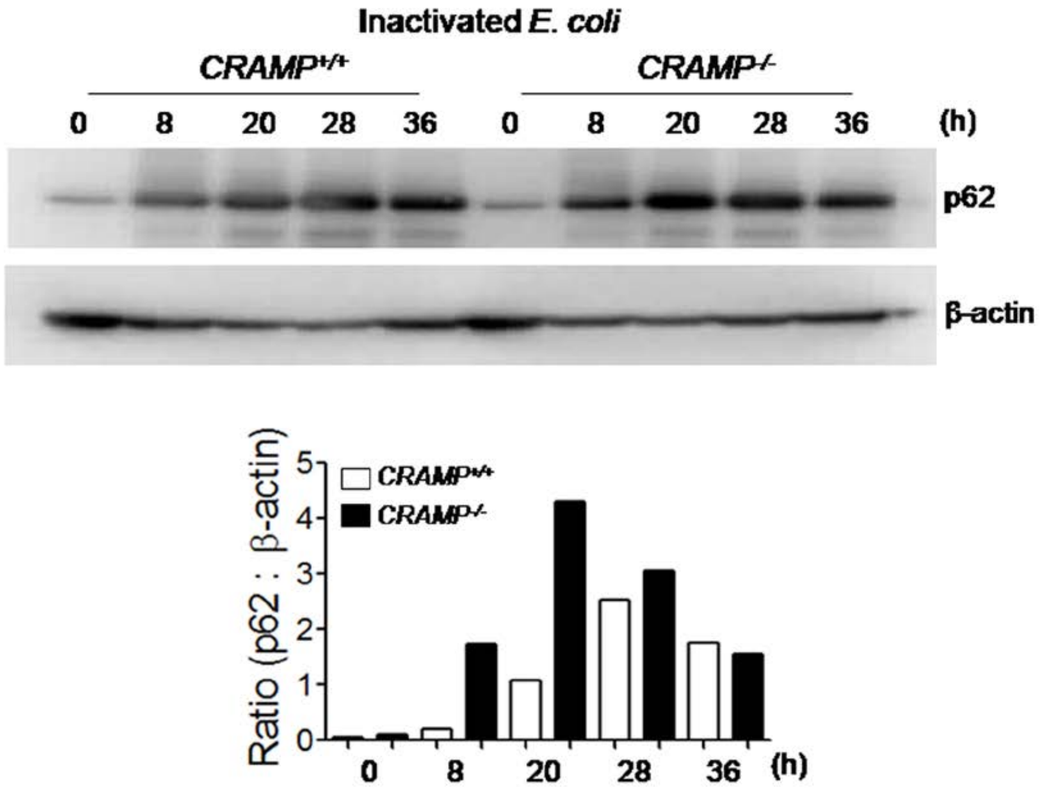

Fig. S3. Delayed degradation of p62 in *CRAMP*$^{-/-}$ macrophages.

BM-derived *CRAMP*$^{+/+}$ control and *CRAMP*$^{-/-}$ macrophages grown in 60-mm dishes to sub-confluency were cultured for 3 h in FCS-free media. The cells were incubated with inactivated *E. coli* (MOI= 10) and harvested at indicated time points followed by lysis. The lysates with titrated proteins were analyzed by Western blotting. For detection of p62, the membranes were incubated with anti-mouse p62 Ab. β-actin was used as a loading control. **Lower panels**: Ratio of p62: β-actin.

Journal of Cell Science • Supplementary information

Table S1. Antibodies and reagents.

REAGENT or RESOURCE

Antibodies	Source	Identifier
Ms mAb to CRAMP	Santa Cruz, TX	Cat# SC-166055
Rb mAb to p62	Abcam, MA	Cat# ab240635
Rb pAb to LC3B	Abcam, MA	Cat# ab48394
Rb mAb to ATG5	Abcam, MA	Cat# ab108327
Anti P-p38 Ab	Cell signaling, MA	Cat# 9211
Anti p38 Ab	Cell signaling, MA	Cat# 9212
Anti P-ERK1/2 Ab	Cell signaling, MA	Cat# 9101
Anti ERK1/2 Ab	Cell signaling, MA	Cat# 9102
Anti P-IκB-α Ab	Cell signaling, MA	Cat# 9241
Anti IκB-α Ab	Cell signaling, MA	Cat# 9242
**Other reagents**		
Mouse CRAMP	Hycult Biotech. PA	Cat# HC1106
Elastatinal	Abcam, MA	Cat# 144541
E64d	Abcam, MA	Cat# 144048
Pepstatin A	Abcam, MA	Cat# 141416
BAY11-7082	Abcam, MA	Cat# 141228
Live/Dead Viability/Cytotoxicity Kit	Them Fisher, MA	Cat# L3224

Journal of Cell Science • Supplementary information
