# Supplementary material for: Requirement of CRAMP for mouse macrophages to eliminate phagocytosed E. coli through an autophagy pathway
Source: J Cell Sci. 2021 Mar 8;134(5):jcs252148. doi: 10.1242/jcs.252148 (PMC7970306; doi:10.1242/jcs.252148)
Supplement: Reviewer comments [file joces-134-252148_review_history.pdf]

Requirement of CRAMP for mouse macrophages to eliminate phagocytosed *E. coli* through an autophagy pathway

Keqiang Chen, Teizo Yoshimura, Wanghua Gong, Cuimeng Tian, Jiaqiang Huang, Giorgio Trinchieri and Ji Ming Wang
DOI: 10.1242/jcs.252148

Editor: Derek Walsh

Review timeline

Original submission:	24 July 2020
Editorial decision:	14 September 2020
First revision received:	20 November 2020
Editorial decision:	9 December 2020
Second revision received:	28 December 2020
Accepted:	30 December 2020

Original submission

First decision letter

MS ID#: JOCES/2020/252148

MS TITLE: Requirement of the antimicrobial peptide CRAMP for macrophages to eliminate phagocytosed E. coli through an autophagy pathway

AUTHORS: Keqiang Chen, Teizo Yoshimura, Wanghua Gong, Cuimeng Tian, Jiaqiang Huang, Giorgio Trinchieri, and Ji-ming Wang
ARTICLE TYPE: Research Article

We have now reached a decision on the above manuscript.

To see the reviewers' reports and a copy of this decision letter, please go to: https://submit-jcs.biologists.org and click on the 'Manuscripts with Decisions' queue in the Author Area. (Corresponding author only has access to reviews.)

As you will see, the reviewers gave favourable reports but raised some critical points that will require amendments to your manuscript. I hope that you will be able to carry these out, because I would like to be able to accept your paper.

We are aware that you may currently be unable to access the lab to undertake experimental revisions. If it would be helpful, we encourage you to contact us to discuss your revision in greater detail. Please send us a point-by-point response indicating where you are able to address concerns raised (either experimentally or by changes to the text) and where you will not be able to do so within the normal timeframe of a revision. We will then provide further guidance. Please also note that we are happy to extend revision timeframes as necessary.

Please ensure that you clearly highlight all changes made in the revised manuscript. Please avoid using 'Tracked changes' in Word files as these are lost in PDF conversion.

I should be grateful if you would also provide a point-by-point response detailing how you have dealt with the points raised by the reviewers in the 'Response to Reviewers' box. Please attend to

© 2021. Published by The Company of Biologists under the terms of the Creative Commons Attribution License (https://creativecommons.org/licenses/by/4.0/).

all of the reviewers' comments. If you do not agree with any of their criticisms or suggestions please explain clearly why this is so.

Reviewer 1

Advance summary and potential significance to field

The manuscript from Ji Ming Wang and colleges studies the role of the antimicrobial peptide CRAMP in the context of macrophages phagocytosis of E.coli. This manuscript demonstrates that CRAMP is expressed when macrophages are challenged with E.coli O22:H8. In addition, they demonstrated that CRAMP is required to inhibit intracellular bacterial replication. And finally, the involvement of autophagy in this process is elucidated. The current understanding of the role of CRAMP during E.coli infection is limited and this manuscript provides novel information on the function of CRAMP in macrophages. With minor corrections, I recommend this manuscript for publication in JCS.

Comments for the author

1. The classical models of E.coli infection involve pathogenic EPEC/EHEC in human or their counterpart C. rodentium in mice. In this work, E.coli O22:H8 is isolated from stools of naïve mice (CRAMP+/+ mice?). Based in literature E.coli O22:H8 mainly produces cattle infection and rarely causes human illness (L. Martorelli et al. Veterinary Microbiology 208 (2017) 8-17). The relevance of E.coli O22:H8 strain needs to be highlighted.
2. Given the existing bibliography about the role of autophagy in E. coli infection should be mention together with Salmonella, Shigella, and Mycobacterium.
3. In the Fig.1A indicates that CRAMP production significantly increases at 20 hpi. But in the Fig.3 A shows that by 20 hpi cells are completely lysed as a consequence of infection. How you rule out that CRAMP production was not higher before 20hpi but the total amount produced was not detected by ELISA because part of CRAMP produced was within the cell. Also, values are only significantly different for 45hpi in the graph when actually they are similar for 20, 30 and 45 hpi. Was this experiment done multiple times? Because error bars are not present in the graph.
4. In the Fig. 1B Left panel: E.coli should be present in the images and the time of infection indicated in the legend. Right panel: How do you determine the area of each macrophage for the quantification? This information is missing. In the legend, it is indicated that 10-16 fields per group were quantified and in the pictures, multiple cells appear in each field. The number of values in the quantification should be bigger than the ones represented (n = 19 for E.coli).
5. In the material and methods for the infection of macrophages, gentamicin was not used for killing the extracellular bacteria. Does it mean that cells were exposed to bacteria for 4, 6, and 24Hs? How do you determine that the bacteria imaged are phagocyted and intracellular?
6. Fig. 1C for the western blot keep the grey background/ black band to be consistent with Fig. 1 F. Add the image of the western blot of fMLF. Information about the LPS used is missing.
7. Fig. 1F In the WB T-IkB-a, the band of 2H of CRAMP-/- is bigger than CRAMP+/+ in the image but in the quantification is the inverse. Instead of Control and KO change it to CRAMP-/- and CRAMP+/+ to be consistent in all figures.
8. Fig. 2A Why the CRAMP+ area/macrophage is used instead Fluorescence intensity of CRAMP+ per macrophage, clarify this inconsistency.
9. Also in the text, it is indicated that the experiment was performed with RAW 264.7 macrophages but in the legend, it says that CRAMP+/+ cells were employed.
10. In the Fig. 2B it should be ug/ml and not ug because the assay was performed in 100ul. The live/dead staining with SYTO9/PI will help to show that CRAMP has damaged the bacterial membrane and this is the cause of the reduce CFU.
11. Fig. 2C One western blot showing that Elastatinal inhibits CRAMP cleavage should be added to this figure.
12. Fig. 3A An staining of live/dead bacteria with SYTO9/PI (propidium Iodide) will be useful here to better show that the reduced number of bacteria in CRAMP+/+ cells is due to the killing of the bacteria by the cells. And the levels of expression of CRAMP in CRAMP-/- and CRAMP+/+ are missing. Instead of using the arrows to show the cell membrane, the limits of the cell should be shown with the dotted line. What black picture means? Is it electron microscopy? What information gives this image that the fluorescence does not show?
The percentage of bacteria+ cells should also be present for E.coli.

© 2021. Published by The Company of Biologists under the terms of the Creative Commons Attribution License (https://creativecommons.org/licenses/by/4.0/).

13. Fig. 5 A and B Was this experiment performed only once? Why there are no error bars in the quantification of the western blot? This is required to validate the difference observed at certain time points. For instance, p62 at 2H.
Also, it is better to compare CRAMP-/- and CRAMP+/+ side by side like in figure 1 F.
14. Fig. 5 C D E To say that the colocalization of bacteria with LAMP1 or p62 is higher or lower the quantification is needed since it is not obvious from the pictures the difference. By 6H in the WB the levels of expression are similar between CRAMP-/- and CRAMP+/+ but the fluoresce intensity is higher for CRAMP-/- at 12h. How do you explain this?

Reviewer 2

Advance summary and potential significance to field

The work submitted by Chen et al. describes the role of CRAMP in the autophagy-mediated elimination of phagocytosed E. coli by macrophages. The depletion of CRAMP resulted in a retention of E. coli. Overall, the manuscript is well written and a interesting for the field. I believe the manuscript is suitable for publication in the journal of cell science, although several revisions are needed.

Comments for the author

Major comments
- In figure 1F, the authors claim that the phosphorylation of IkB-a was diminished in CRAMP-/- macrophages. However, at 6 hours, the phosphorylation of IkB-a increases also in the CRAMP-/- macrophages therefore it seems that the phosphorylation is delayed. The autors should include an extra time point (8h/10h) to show it the increase is continued and reduced just like the control macrophages to show if there is a delay of the phosphorylation.
- In figure 5A and B, the amount of LC3 and p62 is depicted. However only up to 6 hours. If CRAMP is really involved in the autophagy pathway you would expect the levels of CRAMP to be important for the levels of LC3 and p62. However, CRAMP expression is the highest at 20h. Therefore, it would be interesting to include a timepoint of 20h. In addition, quite a strong conclusion is drawn from figure 5B, that p62 expression is reduced at 2h and 4h, however, the difference is very small and with only N=1, no significance can be calculated. This should be repeated to draw such conclusions. This conclusion is also underlined in the discussion at the bottom of page 10. Please proof this or remove the conclusion.

Minor comments:
- Fig 1A different symbols should be used, since the circles of E. coli with the S.E.M. are almost like the squares of the medium.
- Legend of Figure 1C. Macrophages should be with a capital at the start of the sentence.
- Figure 1F, use only one symbol to depict the significant differences, normally * is used.
- Figure 2B in the legend: “…plated on LB agar in triplicates to examine…” indicates that the experiment is performed once but the results of triplicates are depicted. Please repeat the experiment and calculate the statistics on three independent experiments.
- In the result section of “reduced capacity of CRAMP-/- macrophages….” a comment between authors is left in and should be removed.
- Figure 6; the small titles in the figure legend of A, B and C: “A. Upregulation and activation of CRAMP.” etc. are clearer if the are added in the figure. The use a dashed line to distinguish between A, B and C.
- The statistics of figure 1A, 1F should be calculated with the 2-Way ANOVA test including a post-hoc test. A two-tailed Student’s t test is only used comparing the groups together.
- In the legend of figure 6, make sure E. coli is written in italics.
- In the second paragraph of the discussion, please determine whether the macrophages are human or not.
- In the discussion, the authors refer to a paper of Lapaquette et al which states that forced induction of autophagy decreases the numbers of intra-macrophages AIEC and pro-inflammatory cytokine release. Please add some lines in the discussion about the cytokine release in CRAMP-/- macrophages after E. coli infection and how this relate to the findings in this article.

© 2021. Published by The Company of Biologists under the terms of the Creative Commons Attribution License (https://creativecommons.org/licenses/by/4.0/).

Reviewer 3

Advance summary and potential significance to field

The paper by Chen et al describes the role of CRAMP, an antimicrobial peptide in the host defence against E. coli. The authors infect macrophages derived from wild type mice with E. coli and find that CRAMP is upregulated by bacterial infection. Inactivated E. coli and LPS can also induce upregulation of CRAMP in wt mouse macrophages. Further, they show that autophagy is involved in CRAMP-mediated inhibition of bacteria in mouse macrophages. To dissect the role of CRAMP in these processes the authors use CRAMP KO mice and show that autophagy mediated elimination is impaired in CRAMP -/- macrophages. They conclude that CRAMP is required for autophagy mediated clearance of intracellular E. coli in mouse macrophages.

The paper has some merits and describes an interesting mechanism, that has not been investigated in detail for gram negative bacteria. However, the text is can be improved with many typos and comments remaining from the draft version. Thus, a final proof-reading would have been pertinent. In fact, it puts a shadow on the science, which is solid, and gives the impression of a hasty work without attention to detail.

Comments for the author

There are several points that needs to be addressed:
Title: It should be clear from the title that these results only are valid for the mouse-system
Abstract: likewise, the final sentence in the abstract could end with "… in the mouse".
Introduction: The language can be improved with many typos and errors, Please correct.
Intro: the final passage is not needed, instead of providing results, I suggest it is better to describe what the plan was to do, the results will follow in the result-section.
Results: E. coli was obtained from feces of naïve mice. How was this isolate characterized? E. coli can be very different regarding surface structure virulence factors, antibiograms etc. This is important since the general nature of the results may be questioned. How was the heat-inactivation performed?
Results: language can be improved, e.g. "Western blotting", should be "Western blot analysis". Further "observed revealed"… etc Page 7: please, remove the internal comment starting with (K, I moved….)
Results: in general, please who all bacterial killing assays with a CFU-count and not expressed as % or bacteria / cell etc. This is important information for the reader.
Results: how was the bacterial counts in wt and CRAMP -/- macrophages over time? Was there any control of bacterial growth in -/- cells? How large was the difference in bacterial growth between the two different cell-types? This is also key to interpret the magnitude of the findings. Please, provide CFU-counts of the data presented in figure 3B.
Figure 5: Expression of p62 protein in CRAMP +/+ and CRAMP -/- macrophages are similar. During autophagy p62 is going to degrade in autolysosome. But in Figure 5B and 5E looks opposite. In Western Blot analysis there is no difference of p62 expression over the time between in CRAMP +/+ and CRAMP -/- macrophages but confocal microscopy picture follows the hypothesis. Do you have any explanation for these discrepancies?
Discussion: Please, use past tense for your results obtained here.
Remove (human?) on line 12 Please, add a section on the difference between the human and the mouse systems. In fact, human LL-37 is not induced by LPS and has a vitamin D responsive element in the gene promoter. These are important differences between human and mouse. Thus, it is not correct to assume that they are similar in transcription, translation, release, receptor usage or down-stream effects. This should be discussed, I think.

Methods: Heat-inactivation?
In vitro killing assay, please provide the CFU-data.
Did you check the specificity of the ab by performing a western blot analysis on CRAMP -/- cells?

Overall, the paper report an interesting and important story. The experiments are generally well performed, statistical analyses are adequate and results are clear. However, the bacterial

© 2021. Published by The Company of Biologists under the terms of the Creative Commons Attribution License (https://creativecommons.org/licenses/by/4.0/).

experiments can be reported as CFU/ml, which would be important to fully grasp the magnitude of the effects. Finally, the language needs a thorough revision and proof-reading.

First revision

Author response to reviewers' comments

Response to Reviewers:
Reviewer 1: Comments for the author
Q-1. The classical models of E.coli infection involve pathogenic EPEC/EHEC in human or their counterpart C. rodentium in mice. In this work, E.coli O22:H8 is isolated from stools of naïve mice (CRAMP+/+ mice?). Based in literature E.coli O22:H8 mainly produces cattle infection and rarely causes human illness (L. Martorelli et al. Veterinary Microbiology 208 (2017) 8-17). The relevance of E. coli O22:H8 strain needs to be highlighted.

Answer: We thank the Reviewer for the comments.
E. coli strain O22H8 from the feces of mice was identified by whole genome sequencing. O22H8 strain was found in the feces of a variety of mice under different conditions such as naïve and DSS-treated mice in our laboratory. We also verified the potentially innocuous nature of O22H8 as a commensal E. coli. It has been reported that three sub-strains of E. coli O22H8 were isolated from normal healthy cattle, which carry stx1 and stx2d genes and are rarely associated with human illness but in contrast inhibit the expansion of E. coli O157H7 strain that is pathogenic in human by adhering to the colon mucosa to cause bloody diarrhea (Martorelli et al., 2017). Thus, commensal E. coli is beneficial to both human and animal hosts. However, E. coli O22H8 in laboratory mice was rarely reported previously. E. coli belongs to the family of Enterobacteriaceae, phylum of Proteobacteria, which although constitutes a minor fraction of the microbiome found in human gastrointestinal tract (Bailey et al., 2010), is the most common cause of intestinal and extra-intestinal diseases (Conway and Cohen, 2015; Foster, 2004; Katouli, 2010). Many host factors including inflammation and genetic predisposition alter the colonic microbial composition and support the growth of either resident or introduced aerobic bacteria, particularly of the Enterobacteriaceae family (Lupp et al., 2007) such as E. coli elevated in IBD (Bambou et al., 2004; Martin et al., 2004; Rhodes, 2007; Zhang et al., 2017) as well as CRC tissues (Arthur et al., 2012; Dejea et al., 2018). Therefore, it has important clinical significance for investigation of the role of CRAMP in eliminating E. coli by macrophages. We have included the discussions on Page 10, Lines 12-29 of the revised manuscript.

Q-2. Given the existing bibliography about the role of autophagy in E. coli infection should be mention together with Salmonella, Shigella, and Mycobacterium.

Answer: We thank the Reviewer for the comments and suggestions.
It has been reported that LL-37 plays an important role in intracellular bacterial killing by human macrophages. Phenylbutyrate induces LL-37-dependent autophagy and intracellular killing of Mycobacterium tuberculosis in human macrophages (Rekha et al., 2015). Moreover, RNAi-generated mouse CRAMP-/- macrophages and macrophages from CRAMP-/- mouse bone marrow (BM) were significantly impaired in their ability to kill mycobacteria (Sonawane et al., 2011). Another intracellular pathogen Salmonella typhimurium also is inhibited by mouse macrophages depending on intracellular elastase-like serine protease activity to proteolytically activate the LL-37 analogue CRAMP (Rosenberger et al., 2004). Clinical data showed that ileal lesions in Crohn’s disease (CD) patients are abnormally colonized by pathogenic and invasive E. coli (AIEC). AIEC infection of macrophages induces the recruitment of autophagy machinery in the location of phagocytosis to limit intracellular AIEC replication. Impaired ATG16L1, IRGM or NOD2 expression in macrophages increases intracellular AIEC with enhanced secretion of IL-6 and TNF-a in response to infection. In contrast, forced induction of autophagy decreases the numbers of intra-macrophage AIEC and pro-inflammatory cytokine release (Lapaquette et al., 2012). Our study revealed that CRAMP is required for mouse macrophages to kill and eliminate intracellular E. coli, which was supported by the fact that Elastatinal, an elastase inhibitor, attenuated the capacity of macrophages to eliminate

© 2021. Published by The Company of Biologists under the terms of the Creative Commons Attribution License (https://creativecommons.org/licenses/by/4.0/).

phagocytosed E. coli. CRAMP-/- macrophages showed reduced expression of autophagy-related proteins ATG5, LC3-II, LAMP-1 and p62 after phagocytosis of E. coli. These results further support the role of CRAMP-dependent autophagy in the elimination of phagocytosed E. coli by macrophages and in maintaining the colon homeostasis. We have included these statements on Page 11, Lines 14-30 and Page 12, Lines 1-4 of the revised manuscript.

Q-3. In the Fig.1A indicates that CRAMP production significantly increases at 20 hpi. But in the Fig.3 A shows that by 20 hpi cells are completely lysed as a consequence of infection. How you rule out that CRAMP production was not higher before 20hpi but the total amount produced was not detected by ELISA because part of CRAMP produced was within the cell. Also, values are only significantly different for 45hpi in the graph when actually they are similar for 20, 30 and 45 hpi. Was this experiment done multiple times? Because error bars are not present in the graph.

Answer: We thank the Reviewer for the comments.
In Fig. 3A, we used live E. coli to infect both CRAMP+/+ and CRAMP-/- macrophages. CRAMP deficiency in macrophages results in rapid E. coli growth in the cells, which are disintegrated by 20 h. In contrast, CRMAP+/+ macrophages maintained the capacity to kill intracellular E. coli by 20 h, these cells remained intact with only a small number of bacteria visible in the cells.

In Fig. 1A, we used live E. coli to infect CRMAP+/+ macrophages and the production of CRAMP by these cells progressively increased and reached the maximal level by 20 h, then maintained at a stable level until 50 h. CRAMP production by macrophages depends on E. coli infection. After E. coli was killed and eliminated from macrophages, CRAMP production sustained for some time then returned to the normal level after 60 h. We have included these statements on Page 6, Lines 7 of the revised manuscript.

Q-4. In the Fig. 1B Left panel: E.coli should be present in the images and the time of infection indicated in the legend. Right panel: How do you determine the area of each macrophage for the quantification? This information is missing. In the legend, it is indicated that 10-16 fields per group were quantified and, in the pictures, multiple cells appear in each field. The number of values in the quantification should be bigger than the ones represented (n = 19 for E.coli).

Answer: We thank the Reviewer for the comments.
We are sorry for the mistake and have revised as “n = 20-22 macrophages/group” in the Fig. 1B ligand of the revised manuscript.

Q-5. In the material and methods for the infection of macrophages, gentamicin was not used for killing the extracellular bacteria. Does it mean that cells were exposed to bacteria for 4, 6, and 24Hs? How do you determine that the bacteria imaged are phagocyted and intracellular?

Answer: We thank the Reviewer for the reminder.
For live E. coli infection described in Fig. 3A, we have added “Fluorescence staining for macrophage killing of intracellular E. coli” in the Materials and Methods.

BM-derived CRAMP+/+ and CRAMP-/- macrophages seeded in 35 mm dish with 14 mm coverslips in the bottom (MatTeck Corporation, MA) were infected with E. coli O22H8 at a multiplicity of infection of 5 bacteria per cell (MOI=5) at 37ºC in DMEM supplemented with 10% FCS in the presence of M-CSF (50 ng/ml) without antibiotics for 1 h. The cells were then treated with gentamicin (50 μg/ml) for 30 min and washed. The cells were re-cultured and harvested at indicated time points. The cells were fixed in 4% neutral formalin and stained with SYTO 9 (Buisson et al., 2019), after which the number of intracellular bacteria was determined as previously described (Glasser et al., 2001). In contrast, inactivated E. coli was used in most of the experiments. The inactivated bacteria not endocytosed by macrophages were removed with PBS to keep only intracellular bacteria. We have included these statements on Page 15, Lines 1-6 from the bottom and Page 16, Lines 1-3 of the revised manuscript.

We also added the method to inactivate E. coli as follows:
The colony of E. coli O22H8 grown in Violet Red Bile Lactose agar was selected and grown in LB Broth aerobically at 37°C. E. coli was incubated overnight with continuous shaking (200 rpm) in a shaker incubator. The E. coli suspension was diluted to 2 × 106 CFU/ml and 0.4% formalin (by

© 2021. Published by The Company of Biologists under the terms of the Creative Commons Attribution License (https://creativecommons.org/licenses/by/4.0/).

volume ratio) was added for incubation overnight with continuous shaking (200 rpm) at 37°C. A small amount of inactivated E. coli (5 µl for each sample) was used to culture in Violet Red Bile Lactose agar dishes in an incubator at 37°C overnight. No E. coli growth was observed (Landman and van Eck, 2017). We have included these statements on Page 14, Lines 2-9 of the revised manuscript.

Q-6. Fig. 1C for the western blot keep the grey background/ black band to be consistent with Fig. 1 F. Add the image of the western blot of fMLF. Information about the LPS used is missing.

Answer: We thank the Reviewer for the suggestion.
1. We have converted the grey background/black band of Fig. 1C to be consistent with Fig. 1F.
2. We used ELISA to measure CRAMP production induced by LPS (Fig. 1D) and fMLF (Fig. 1E) instead of Western blot.

Q-7. Fig. 1F In the WB T-IkB-a, the band of 2H of CRAMP-/- is bigger than CRAMP+/+ in the image but in the quantification is the inverse. Instead of Control and KO change it to CRAMP-/- and CRAMP+/+ to be consistent in all figures.

Answer: We thank the Reviewer for pointing out the mistake. We have re-measured the data and revised the figure.

Q-8. Fig. 2A Why the CRAMP+ area/macrophage is used instead Fluorescence intensity of CRAMP+ per macrophage, clarify this inconsistency.

Answer: We thank the Reviewer for the suggestion. We have re-analyzed the results and used Fluorescence intensity of CRAMP per macrophage to replace CRAMP positive area/macrophage.

Q-9. Also in the text, it is indicated that the experiment was performed with RAW 264.7 macrophages but in the legend, it says that CRAMP+/+ cells were employed.

Answer: We thank the Reviewer for pointing out the mistake. We have corrected the mistake.

Q-10. In the Fig. 2B it should be ug/ml and not ug because the assay was performed in 100ul.
The live/dead staining with SYTO9/PI will help to show that CRAMP has damaged the bacterial membrane and this is the cause of the reduce CFU.

Answer: We thank the Reviewer for pointing out the mistake, which we have corrected.

Q-11. Fig. 2C One western blot showing that Elastatinal inhibits CRAMP cleavage should be added to this figure.

Answer: We thank the Reviewer for the suggestion. Although it is a valid idea to use Western blot to show that Elastatinal inhibits CRAMP cleavage, we were unable to perform the experiment, since a specific antibody against mature CRAMP was not available. Mature CRAMP is released from its precursor by proteinase (Sorensen et al., 2001) or elastase (Gudmundsson et al., 1996; Molla et al., 1993). Abcam company provides an anti-Cathelicidin/CLP antibody (Cat #: ab180760), which reacts with mouse CRAMP and human LL-37 only suitable for WB and IHC-P. The antibody was generated with recombinant full-length protein corresponding to human Cathelicidin/CLP aa 31-170 as an immunogen. Therefore, the antibody is not able to recognize the mature mouse CRAMP peptide. Another antibody from Santa Cruz (Cat #: sc-166055) is a mouse monoclonal antibody raised against amino acids 6-175 in the C-terminus of CRAMP of rat origin and reacts with CRAMP of mouse and rat origin by Western blotting but was unable to recognize mature mouse CRAMP peptide. Elastatinal used in this study was documented to inhibit the elastase in neutrophils (Gilbert et al., 1997), microglia (Nakajima et al., 1992) and macrophages (Rosenberger et al., 2004) as supported by our result in Fig. 2C.

Q-12. Fig. 3A An staining of live/dead bacteria with SYTO9/PI (propidium Iodide) will be useful here to better show that the reduced number of bacteria in CRAMP+/+ cells is due to the killing of the bacteria by the cells. And the levels of expression of CRAMP in CRAMP-/- and CRAMP+/+ are missing. Instead of using the arrows to show the cell membrane, the limits of the cell should be

© 2021. Published by The Company of Biologists under the terms of the Creative Commons Attribution License (https://creativecommons.org/licenses/by/4.0/).

shown with the dotted line. What black picture means? Is it electron microscopy? What information gives this image that the fluorescence does not show? The percentage of bacteria+ cells should also be present for E. coli.

Answer: We thank the Reviewer for the comments and suggestions.
1). CRAMP+/+ macrophages were derived from CRAMP+/+ mice (WT mice) and they express CRAMP. In contrast, CRAMP-/- macrophages were derived from Myeloid CRAM/-/- mice, which were deficient in CRAMP. Now we have added the data of CRAMP expression in CRAMP+/+ and CRAMP-/- macrophages in Supplemental Fig. 1 of the revised manuscript.
2). The normal black/white picture reproduced the results of fluorescence shown at 4 h after E. coli infection.
3). The percentage of bacteria+ cells was not shown because some macrophages were fragmentated after E. coli replication. Therefore, it is not possible to count the exact percentage of bacteria+ cells.

Q-13. Fig. 5 A and B Was this experiment performed only once? Why there are no error bars in the quantification of the western blot? This is required to validate the difference observed at certain time points. For instance, p62 at 2H. Also, it is better to compare CRAMP-/- and CRAMP+/+ side by side like in figure 1 F.

Answer: We thank the Reviewer for the reminder. We have revised these figures according to the Reviewer's suggestions.

Q-14. Fig. 5 C D E To say that the colocalization of bacteria with LAMP1 or p62 is higher or lower the quantification is needed since it is not obvious from the pictures the difference. By 6H in the WB the levels of expression are similar between CRAMP-/- and CRAMP+/+ but the fluoresce intensity is higher for CRAMP-/- at 12h. How do you explain this?

Answer: We thank the Reviewer for the comments. The role of p62(A170/SQSTM1) in autophagy is not fully understood. Some reported functions are as follows (Ichimura et al., 2008): 1). p62 is involved in inclusion body formation when macrophages phagocytose bacteria. p62/A170/SQSTM1, a ubiquitin-binding protein, participates in inclusion body formation during phagocytosis process of the cells. 2). p62 interacts with LC3 which regulates autophagosome formation. p62 delivers specific cytosolic components, including ribosomal protein S30 (rpS30) and additional ubiquitinated proteins, to autophagic organelles and interacts with LC3 through a 11 amino acid sequence rich in acidic and hydrophobic residues, named LC3-recognition sequence (LRS). 3). LC3-p62 complex is eventually degraded in autolysosome. In the absence of p62, the cells are unable to generate neo-antibacterial factors, resulting in non-functional autophagy despite maturation, thereby failing to effectively eliminate intracellular bacteria (Ponpuak et al., 2010). The degradation of p62 is a widely used parameter to monitor autophagic activity because p62 binds to LC3 and is selectively degraded during autophagy (Bjorkoy et al., 2005; Pankiv et al., 2007). In our study, CRAMP deficiency reduced the expression of p62 by mouse macrophages when inactivated E. coli was phagocytized. After 6 h, p62 expression was significantly increased, indicating that the dead E. coli included in p62 complex accumulated in autolysosomes. These data suggest that the autophagic process in macrophages to eliminate intracellular bacteria was impaired in the absence of CRAMP. We have included these statements on Page 12, Lines 3-20 of the revised manuscript.

In this manuscript, Fig. 6 does not contain panel H.

Reviewer 2: Comments for the author
Major comments
Q-1. In figure 1F, the authors claim that the phosphorylation of IkB-a was diminished in CRAMP-/- macrophages. However, at 6 hours, the phosphorylation of IkB-a increases also in the CRAMP-/- macrophages, therefore it seems that the phosphorylation is delayed. The authors should include an extra time point (8h/10h) to show it the increase is continued and reduced just like the control macrophages to show if there is a delay of the phosphorylation.

Answer: We thank the Reviewer for the comments and suggestions. We have amended with the following statements:

© 2021. Published by The Company of Biologists under the terms of the Creative Commons Attribution License (https://creativecommons.org/licenses/by/4.0/).

As shown in Fig. 1F, the intensity of phosphorylation of IκB-α induced by inactivated E. coli at 1 h and 2 h was significantly higher in CRAMP+/+ macrophages than in CRAMP-/- macrophages. At 6 h, the phospho-IκB-α began to increase again but there was no significant difference between CRAMP+/+ and CRAMP-/- macrophages. Also, the intensity of de novo synthesis of total IκB-α was higher at 2 h after stimulation with inactivated E. coli in CRAMP+/+ macrophages than in CRAMP-/- macrophages. We have included these statements on Page 6, Lines 16-24 of the revised manuscript.

Q-2. In figure 5A and B, the amount of LC3 and p62 is depicted. However, only up to 6 hours. If CRAMP is really involved in the autophagy pathway, you would expect the levels of CRAMP to be important for the levels of LC3 and p62. However, CRAMP expression is the highest at 20h. Therefore, it would be interesting to include a timepoint of 20h. In addition, quite a strong conclusion is drawn from figure 5B, that p62 expression is reduced at 2h and 4h, however, the difference is very small and with only N=1, no significance can be calculated. This should be repeated to draw such conclusions. This conclusion is also underlined in the discussion at the bottom of page 10. Please proof this or remove the conclusion.

Answer: We thank the Reviewer for the comments and suggestions.
1. We modified Fig. 5A-B by adding reproduced results in the revised manuscript.
2. We also added new results showing that p62 expression is higher in CRAMP-/- macrophages than in CRAMP+/+ macrophages at 8, 20, 28 and 36 h after stimulation with inactivated E. coli (Supplemental Fig. 3 of the revised manuscript), indicating that the dead E. coli included in p62 complex accumulated in autolysosomes of CRAMP-/- macrophages.

Minor comments:
Q-3. Fig 1A different symbols should be used, since the circles of E. coli with the S.E.M. are almost like the squares of the medium.

Answer: We thank the Reviewer for the comments. We have modified Fig. 1A.

Q-4. Legend of Figure 1C. Macrophages should be with a capital at the start of the sentence.
-Figure 1F, use only one symbol to depict the significant differences, normally * is used.

Answer: We thank the Reviewer for the reminder of corrections.

Q-5. Figure 2B in the legend: "…plated on LB agar in triplicates to examine…" indicates that the experiment is performed once but the results of triplicates are depicted. Please repeat the experiment and calculate the statistics on three independent experiments.

Answer: We thank the Reviewer for the suggestion with modifications in Fig. 2B.

Q-6. In the result section of "reduced capacity of CRAMP-/- macrophages…." a comment between authors is left in and should be removed.

Answer: We are sorry for the error and thank the Reviewer for the reminder. We have removed the comment.

Q-7. Figure 6; the small titles in the figure legend of A, B and C: "A. Upregulation and activation of CRAMP." etc. are clearer if the are added in the figure. The use a dashed line to distinguish between A, B and C.

Answer: We thank the Reviewer for the suggestion. We have modified Fig. 6 and added the subtitles of A, B and C as well as dashed lines to distinguish A, B and C.

Q-8. The statistics of figure 1A, 1F should be calculated with the 2-Way ANOVA test including a post-hoc test. A two-tailed Student's t test is only used comparing the groups together.

Answer: We thank the Reviewer for the reminder. We used ANOVA including a post-hoc test.

Q-9. In the legend of figure 6, make sure E. coli is written in italics.

© 2021. Published by The Company of Biologists under the terms of the Creative Commons Attribution License (https://creativecommons.org/licenses/by/4.0/).

Answer: We thank the Reviewer for the reminder. We have made sure of using italics for E. coli all over.

Q-10. In the second paragraph of the discussion, please determine whether the macrophages are human or not.

Answer: We thank the Reviewer for the reminder. We have revised the statement on Page 11, Lines 1-13 of the revised manuscript.

Q-11. In the discussion, the authors refer to a paper of Lapaquette et al which states that forced induction of autophagy decreases the numbers of intra-macrophages AIEC and pro-inflammatory cytokine release. Please add some lines in the discussion about the cytokine release in CRAMP-/- macrophages after E. coli infection and how this relate to the findings in this article.

Answer: We thank the Reviewer for the suggestion. We have added the following description in the Discussion:
Cytokines are potent signaling molecules as important as hormones and neurotransmitters. When macrophages are exposed to inflammatory stimuli, they secrete cytokines such as tumor necrosis factor (TNF), IL-1, IL-6, IL-8, and IL-12 (Arango Duque and Descoteaux, 2014). In the gut, macrophages residing in the mucosa are able to prevent the entry and colonization of pathogens in the mucosa (Weiss and Schaible, 2015). In inflamed gut, inflammatory macrophages are sequentially recruited to mount appropriate immune responses and produce cytokines (Na et al., 2019). However, autophagy deficiency in macrophages increased not only the survival of intra-cellular bacteria, but also the secretion of pro-inflammatory cytokines. The gut lesions in Crohn's disease (CD) patients are abnormally colonized by pathogenic adherent/invasive E. coli (AIEC). In infected macrophages, AIEC bacteria induced the recruitment of the autophagy machinery at the site of phagocytosis, and functional autophagy limited intra-cellular AIEC replication. Impaired ATG16L1, IRGM or NOD2 expression increases in intra-cellular AIEC and secretion of IL-6 and TNF-a by macrophages in response to AIEC infection. In contrast, forced induction of autophagy decreased the numbers of intra-macrophagic AIEC and pro-inflammatory cytokine release, even in a NOD2-deficient context (Lapaquette et al., 2012). Another study also showed that macrophages defect in mediating AIEC clearance and increasing pro-inflammatory cytokines IL-1β and TNF-α in CD patients are linked to polymorphisms related to autophagy such as IRGM and ULK-1 (Buisson et al., 2019). In vivo, CRAMP-/- mice showed increased susceptibility to Pseudomonas aeruginosa (PA) keratitis and enhanced secretion of pro-inflammatory cytokines including IL-1β, IL-6 and TNF-α in PA-infected corneas (Huang et al., 2007). Our present study showed that active CRAMP was required for macrophages to eliminate phagocytosed E. coli, with participation of autophagy-related proteins ATG5, LC3-II, and LAMP-1 as well as conjugation of the bacteria with p62. In addition, myeloid CRAMP-/- mice, but not epithelial CRAMP-/- mice, showed increased plasma levels of IL-1β and IL-6 after DSS treatment for 5 d (Chen et al., 2020). Based on these results, we hypothesize that stimulating autophagy machinery in macrophages in IBD patients could be a plausible therapeutic strategy to concomitantly restraining intracellular bacterial replication and dampening inflammatory responses. We have included these statements on Page12, Lines 1-4 from bottom and Page 13, Lines 1-24 of the revised manuscript.

Reviewer 3: Comments for the author
Q-1. Title: It should be clear from the title that these results only are valid for the mouse-system

Answer: We thank the Reviewer for the comments and suggestions. We have amended as follows:
Requirement of the antimicrobial peptide CRAMP for mouse macrophages to eliminate phagocytosed E. coli through an autophagy pathway.

Q-2. Abstract: likewise, the final sentence in the abstract could end with "… in the mouse".

Answer: We thank the Reviewer for the comments and suggestions. We have amended as follows:

© 2021. Published by The Company of Biologists under the terms of the Creative Commons Attribution License (https://creativecommons.org/licenses/by/4.0/).

These results indicate CRAMP as a critical component in autophagy-mediated clearance of intracellular E. coli by mouse macrophages. We have included these changes on Page 2, Lines 13-14 of the revised manuscript.

Q-3. Introduction: The language can be improved with many typos and errors, Please correct. Intro: the final passage is not needed, instead of providing results, I suggest it is better to describe what the plan was to do, the results will follow in the result-section.

Answer: We thank the Reviewer for the comments and suggestions.
1. We have checked and corrected typos and errors in the Introduction to the best of our knowledge.
2. We have also amended the last passage in introduction as follows:
In this study, we investigated the expression of CRAMP in mouse macrophages after stimulation with E. coli and the role of CRAMP in the elimination of intracellular inactivated E. coli by using macrophages derived from the bone marrow (BM) of CRAMP-/- mice. Furthermore, we explored the relationship between CRAMP and autophagy processes in mouse macrophages. We have included these statements on Page 5, Lines 5-8 of the revised manuscript.

Q-4. Results: E. coli was obtained from feces of naïve mice. How was this isolate characterized? E. coli can be very different regarding surface structure, virulence factors, antibiograms etc. This is important since the general nature of the results may be questioned. How was the heat-inactivation performed?

Answer: We thank the Reviewer for the comments and suggestions. We have added the following description in the Discussion section of the revised manuscript:
E. coli strain O22H8 from the feces of mice was identified by whole genome sequencing. O22H8 strain was found in the feces of a variety of mice under different conditions such as naïve and DSS-treated mice as seen in our study. Our study also verified the potentially innocuous nature of O22H8 as a commensal E. coli. It has been reported that three sub-strains of E. coli O22H8 were isolated from normal healthy cattle, which carry stx1 and stx2d genes and are rarely associated with human illness but in contrast inhibit the expansion of E. coli O157H7 strain that is pathogenetic in human by adhering to colon mucosa to cause bloody diarrhea (Martorelli et al., 2017). Thus, commensal E. coli is beneficial to both human and animal hosts. However, E. coli O22H8 is rarely reported in laboratory mice previously. E. coli belongs to the family of Enterobacteriaceae, phylum of Proteobacteria, which although constitutes a minor fraction of the microbiome found in human gastrointestinal tract (Bailey et al., 2010), is the most common cause of intestinal and extra-intestinal diseases (Conway and Cohen, 2015; Foster, 2004; Katouli, 2010). Many host factors including inflammation and genetic predisposition alter the colonic microbial composition and support the growth of either resident or introduced aerobic bacteria, particularly Enterobacteriaceae family (Lupp et al., 2007) such as E. coli that is elevated in IBD (Bambou et al., 2004; Martin et al., 2004; Rhodes, 2007; Zhang et al., 2017) as well as in CRC tissues (Arthur et al., 2012; Dejea et al., 2018). Therefore, it is important to investigate the role of CRAMP in eliminating E. coli by macrophages. We have included these sentences on Page 10, Lines 12-29 of the revised manuscript.

Q-5. Results: language can be improved, e.g. “Western blotting”, should be “Western blot analysis”. Further “observed revealed”, etc

Answer: We thank the Reviewer for the comments and suggestions. We have checked through the manuscript and tried our best to improve English writing quality.

Q-6. Page 7: please, remove the internal comment starting with (K, I moved….)
Results: in general, please who all bacterial killing assays with a CFU-count and not expressed as % or bacteria / cell etc. This is important information for the reader.

Answer: We are sorry for the error and thank the Reviewer for the reminder.
1. We have removed the comment in the text.
2. We used bacteria: cells ration based on Multiplicity of infection (MOI)-Wikipedia (https://en.wikipedia.org/wiki/Multiplicity_of_infection). MOI is the ratio of bacteria to cells.

© 2021. Published by The Company of Biologists under the terms of the Creative Commons Attribution License (https://creativecommons.org/licenses/by/4.0/).

In our experiments, we first obtained the CFU/ml based on OD600 nm = 0.4 corresponding to ~2 × 108 colony forming units (cfu)/ml. We then calculated the ratio of bacteria: cells (MOI) for infection (live bacteria) or for co-culture with cells (inactivated bacteria).

3. The inactivated E. coli was used in most of our experiments to compare the capacity of macrophages to eliminate phagocytized bacteria by macrophages from CRAMP+/+ and CRAMP-/- mice. Inactivated E. coli cannot be cultured in LB to obtain CFU, it is therefore expressed as the percentage of cells containing bacteria and the number of bacteria in each cell.

Q-7. Results: how was the bacterial counts in wt and CRAMP-/- macrophages over time? Was there any control of bacterial growth in-/- cells? How large was the difference in bacterial growth between the two different cell-types? This is also key to interpret the magnitude of the findings. Please, provide CFU-counts of the data presented in figure 3B.

Answer: We thank the Reviewer for the comment and suggestion.
1. In Fig. 3B, we used inactivated E. coli labeled with FITC to co-culture with mouse macrophages. Inactivated bacteria will not grow in culture to form CFU.
2. In Fig. 3A, we used live E. coli labeled with FITC to infect CRAMP+/+ and CRAMP-/- macrophages to obtain the percentage of bacteria infected cells and the bacterial intensity per cell.
3. We added a new experiment “LB agar incubation to measure macrophage killing of intracellular E. coli”. We show that CRAMP-/- macrophages infected with E. coli, after treatment with gentamicin to eliminate extracellular E. coli and then cultured in the presence of gentamicin for 20 h, contained higher number of intracellular E. coli as compared with CRAMP+/+ macrophages (Supplementary Fig. 2).
We have included these sentences on Page 7, Lines 21-24 and Page 16, Lines 4-16 of the revised manuscript.

Q-8. Figure 5: Expression of p62 protein in CRAMP +/+ and CRAMP -/- macrophages are similar. During autophagy p62 is going to degrade in autolysosome. But in Figure 5B and 5E looks opposite. In Western Blot analysis there is no difference of p62 expression over the time between in CRAMP +/+ and CRAMP -/- macrophages but confocal microscopy picture follows the hypothesis. Do you have any explanation for these discrepancies?

Answer: We thank the Reviewer for the comment.
The role of p62(A170/SQSTM1) in autophagy is not fully understood. Some reported functions are as follows (Ichimura et al., 2008):
1). p62 is involved in inclusion body formation when macrophages phagocytize bacteria. p62, a ubiquitin-binding protein, participates in inclusion body formation during phagocytosis.
2). p62 interacts with LC3 which regulates autophagosome formation. p62 delivers specific cytosolic components, including ribosomal protein S30 (rpS30) and additional ubiquitinated proteins, to autophagic organelles and interacts with LC3 through a 11 amino acid sequence rich in acidic and hydrophobic residues, named LC3-recognition sequence (LRS).
3). LC3-p62 complex eventually is degraded in autolysosome.
In our study, CRAMP deficiency reduced the expression of p62 by mouse macrophages when inactivated E. coli was phagocytized. After 6 h (8, 20, 28 h), p62 expression was significantly increased, indicating that the inactivated E. coli included in p62 complex accumulated in autolysosomes. These data suggest that the autophagic process in macrophages to eliminate intracellular bacteria was impaired in the absence of CRAMP. We have included these statements on Page 12, Lines 7-24, and Supplementary Fig. 3 of the revised manuscript.

Q-9. Discussion: Please, use past tense for your results obtained here.
Remove (human?) on line 12 Please, add a section on the difference between the human and the mouse systems. In fact, human LL-37 is not induced by LPS and has a vitamin D responsive element in the gene promoter. These are important differences between human and mouse. Thus, it is not correct to assume that they are similar in transcription, translation, release, receptor usage or down-stream
effects. This should be discussed, I think.

Answer: We thank the Reviewer for the suggestions.
We have used past tense for our results and amended the Discussion to distinguish LL-37 in humans from CRAMP in mice in the revised manuscript.

© 2021. Published by The Company of Biologists under the terms of the Creative Commons Attribution License (https://creativecommons.org/licenses/by/4.0/).

Q-10. Methods: Heat-inactivation?
In vitro killing assay, please provide the CFU-data.
Did you check the specificity of the ab by performing a western blot analysis on CRAMP-/- cells?

Answer: We thank the Reviewer for the comment and suggestion.
1. We have added the protocol of Heat-inactivation for E. coli on Page 15, Lines 2-8 of the revised manuscript.
2. We have revised Fig. 2B by providing the CFU-data for CRAMP to kill E. coli in vitro.
3. CRAMP-/- macrophages were derived from BM of CRAMP-/- mice. We verified each CRAMP-/- mouse by PCR genotyping to confirm the deletion of Cnlp gene. We also added a new experiment to show reduced CRAMP expression in CRAMP-/- macrophages. We have included these statements on Page 7, Lines 12-14 and Supplemental Fig. 1 of the revised manuscript.

Q-11. Overall, the paper report an interesting and important story. The experiments are generally well performed, statistical analyses are adequate and results are clear. However, the bacterial experiments can be reported as CFU/ml, which would be important to fully grasp the magnitude of the effects. Finally, the language needs a thorough revision and proof-reading.

Answer: We thank the Reviewer for the comments. We hope our revised manuscript, thoroughly revised based on the comments and suggestions, is now suitable for publication in the Journal of Cell Sciences.

References

Arango Duque, G. and Descoteaux, A. (2014). Macrophage cytokines: involvement in immunity and infectious diseases. Front Immunol 5, 491.

Arthur, J. C., Perez-Chanona, E., Muhlbauer, M., Tomkovich, S., Uronis, J. M., Fan, T. J., Campbell, B. J., Abujamel, T., Dogan, B., Rogers, A. B. et al. (2012). Intestinal inflammation targets cancer-inducing activity of the microbiota. Science 338, 120-3.

Bailey, J. K., Pinyon, J. L., Anantham, S. and Hall, R. M. (2010). Commensal Escherichia coli of healthy humans: a reservoir for antibiotic-resistance determinants. J Med Microbiol 59, 1331-9.

Bambou, J. C., Giraud, A., Menard, S., Begue, B., Rakotobe, S., Heyman, M., Taddei, F., Cerf-Bensussan, N. and Gaboriau-Routhiau, V. (2004). In vitro and ex vivo activation of the TLR5 signaling pathway in intestinal epithelial cells by a commensal Escherichia coli strain. J Biol Chem 279, 42984-92.

Bjorkoy, G., Lamark, T., Brech, A., Outzen, H., Perander, M., Overvatn, A., Stenmark, H. and Johansen, T. (2005). p62/SQSTM1 forms protein aggregates degraded by autophagy and has a protective effect on huntingtin-induced cell death. J Cell Biol 171, 603-14.

Buisson, A., Douadi, C., Ouchchane, L., Goutte, M., Hugot, J. P., Dubois, A., Minet-Quinard, R., Bouvier, D., Bommelaer, G., Vazeille, E. et al. (2019). Macrophages Inability to Mediate Adherent-Invasive E. coli Replication is Linked to Autophagy in Crohn's Disease Patients. Cells 8.

Chen, K., Yoshimura, T., Yao, X., Gong, W., Huang, J., Dzutsev, A. K., McCulloch, J., O'HUigin, C., Bian, X. W., Trinchieri, G. et al. (2020). Distinct contributions of cathelin-related antimicrobial peptide (CRAMP) derived from epithelial cells and macrophages to colon mucosal homeostasis. J Pathol.

Conway, T. and Cohen, P. S. (2015). Commensal and Pathogenic Escherichia coli Metabolism in the Gut. Microbiol Spectr 3.

Dejea, C. M., Fathi, P., Craig, J. M., Boleij, A., Taddese, R., Geis, A. L., Wu, X., DeStefano Shields, C. E., Hechenbleikner, E. M., Huso, D. L. et al. (2018). Patients with familial adenomatous polyposis harbor colonic biofilms containing tumorigenic bacteria. Science 359, 592-597.

Foster, J. W. (2004). Escherichia coli acid resistance: tales of an amateur acidophile. Nat Rev Microbiol 2, 898-907.

Gilbert, S. J., Wotton, P. R., Tarlton, J. F., Duance, V. C. and Bailey, A. J. (1997). Increased expression of promatrix metalloproteinase-9 and neutrophil elastase in canine dilated cardiomyopathy. Cardiovasc Res 34, 377-83.

Glasser, A. L., Boudeau, J., Barnich, N., Perruchot, M. H., Colombel, J. F. and Darfeuille-Michaud, A. (2001). Adherent invasive Escherichia coli strains from patients with Crohn's disease

© 2021. Published by The Company of Biologists under the terms of the Creative Commons Attribution License (https://creativecommons.org/licenses/by/4.0/).

survive and replicate within macrophages without inducing host cell death. Infect Immun 69, 5529-37.

Gudmundsson, G. H., Agerberth, B., Odeberg, J., Bergman, T., Olsson, B. and Salcedo, R. (1996). The human gene FALL39 and processing of the cathelin precursor to the antibacterial peptide LL-37 in granulocytes. Eur J Biochem 238, 325-32.

Huang, L. C., Reins, R. Y., Gallo, R. L. and McDermott, A. M. (2007). Cathelicidin-deficient (Cnlp -/-) mice show increased susceptibility to Pseudomonas aeruginosa keratitis. Invest Ophthalmol Vis Sci 48, 4498-508.

Ichimura, Y., Kominami, E., Tanaka, K. and Komatsu, M. (2008). Selective turnover of p62/A170/SQSTM1 by autophagy. Autophagy 4, 1063-6.

Katouli, M. (2010). Population structure of gut Escherichia coli and its role in development of extra-intestinal infections. Iran J Microbiol 2, 59-72.

Landman, W. J. M. and van Eck, J. H. H. (2017). The efficacy of inactivated Escherichia coli autogenous vaccines against the E. coli peritonitis syndrome in layers. Avian Pathol 46, 658-665.

Lapaquette, P., Bringer, M. A. and Darfeuille-Michaud, A. (2012). Defects in autophagy favour adherent-invasive Escherichia coli persistence within macrophages leading to increased pro-inflammatory response. Cell Microbiol 14, 791-807.

Lupp, C., Robertson, M. L., Wickham, M. E., Sekirov, I., Champion, O. L., Gaynor, E. C. and Finlay, B. B. (2007). Host-mediated inflammation disrupts the intestinal microbiota and promotes the overgrowth of Enterobacteriaceae. Cell Host Microbe 2, 119-29.

Martin, H. M., Campbell, B. J., Hart, C. A., Mpofu, C., Nayar, M., Singh, R., Englyst, H., Williams, H. F. and Rhodes, J. M. (2004). Enhanced Escherichia coli adherence and invasion in Crohn's disease and colon cancer. Gastroenterology 127, 80-93.

Martorelli, L., Albanese, A., Vilte, D., Cantet, R., Bentancor, A., Zolezzi, G., Chinen, I., Ibarra, C., Rivas, M., Mercado, E. C. et al. (2017). Shiga toxin-producing Escherichia coli (STEC) O22:H8 isolated from cattle reduces E. coli O157:H7 adherence in vitro and in vivo. Vet Microbiol 208, 8-17.

Molla, A., Hellen, C. U. and Wimmer, E. (1993). Inhibition of proteolytic activity of poliovirus and rhinovirus 2A proteinases by elastase-specific inhibitors. J Virol 67, 4688-95.

Na, Y. R., Stakenborg, M., Seok, S. H. and Matteoli, G. (2019). Macrophages in intestinal inflammation and resolution: a potential therapeutic target in IBD. Nat Rev Gastroenterol Hepatol 16, 531-543.

Nakajima, K., Shimojo, M., Hamanoue, M., Ishiura, S., Sugita, H. and Kohsaka, S. (1992). Identification of elastase as a secretory protease from cultured rat microglia. J Neurochem 58, 1401-8.

Pankiv, S., Clausen, T. H., Lamark, T., Brech, A., Bruun, J. A., Outzen, H., Overvatn, A., Bjorkoy, G. and Johansen, T. (2007). p62/SQSTM1 binds directly to Atg8/LC3 to facilitate degradation of ubiquitinated protein aggregates by autophagy. J Biol Chem 282, 24131-45.

Ponpuak, M., Davis, A. S., Roberts, E. A., Delgado, M. A., Dinkins, C., Zhao, Z., Virgin, H. W. t., Kyei, G. B., Johansen, T., Vergne, I. et al. (2010). Delivery of cytosolic components by autophagic adaptor protein p62 endows autophagosomes with unique antimicrobial properties. Immunity 32, 329-41.

Rekha, R. S., Rao Muvva, S. S., Wan, M., Raqib, R., Bergman, P., Brighenti, S., Gudmundsson, G. H. and Agerberth, B. (2015). Phenylbutyrate induces LL-37-dependent autophagy and intracellular killing of Mycobacterium tuberculosis in human macrophages. Autophagy 11, 1688-99.

Rhodes, J. M. (2007). The role of Escherichia coli in inflammatory bowel disease. Gut 56, 610-2.

Rosenberger, C. M., Gallo, R. L. and Finlay, B. B. (2004). Interplay between antibacterial effectors: a macrophage antimicrobial peptide impairs intracellular Salmonella replication. Proc Natl Acad Sci U S A 101, 2422-7.

Sonawane, A., Santos, J. C., Mishra, B. B., Jena, P., Progida, C., Sorensen, O. E., Gallo, R., Appelberg, R. and Griffiths, G. (2011). Cathelicidin is involved in the intracellular killing of mycobacteria in macrophages. Cell Microbiol 13, 1601-17.

Sorensen, O. E., Follin, P., Johnsen, A. H., Calafat, J., Tjabringa, G. S., Hiemstra, P. S. and Borregaard, N. (2001). Human cathelicidin, hCAP-18, is processed to the antimicrobial peptide LL-37 by extracellular cleavage with proteinase 3. Blood 97, 3951-9.

Weiss, G. and Schaible, U. E. (2015). Macrophage defense mechanisms against intracellular bacteria. Immunol Rev 264, 182-203.

© 2021. Published by The Company of Biologists under the terms of the Creative Commons Attribution License (https://creativecommons.org/licenses/by/4.0/).

Zhang, S. L., Wang, S. N. and Miao, C. Y. (2017). Influence of Microbiota on Intestinal Immune System in Ulcerative Colitis and Its Intervention. Front Immunol 8, 1674.
/

Second decision letter

MS ID#: JOCES/2020/252148

MS TITLE: Requirement of CRAMP for mouse macrophages to eliminate phagocytosed E. coli through an autophagy pathway

AUTHORS: Keqiang Chen, Teizo Yoshimura, Wanghua Gong, Cuimeng Tian, Jiaqiang Huang, Giorgio Trinchieri, and Ji-ming Wang
ARTICLE TYPE: Research Article

We have now reached a decision on the above manuscript. As you will see, all three reviewers gave favourable reports but requested some minor clarifications and changes in the text.I hope that you will be able to carry these out, because I would like to be able to accept your paper.

To see the reviewers' reports and a copy of this decision letter, please go to: https://submit-jcs.biologists.org and click on the 'Manuscripts with Decisions' queue in the Author Area. (Corresponding author only has access to reviews.)

We are aware that you may be experiencing disruption to the normal running of your lab that makes experimental revisions challenging. If it would be helpful, we encourage you to contact us to discuss your revision in greater detail. Please send us a point-by-point response indicating where you are able to address concerns raised (either experimentally or by changes to the text) and where you will not be able to do so within the normal timeframe of a revision. We will then provide further guidance. Please also note that we are happy to extend revision timeframes as necessary.

Please ensure that you clearly highlight all changes made in the revised manuscript. Please avoid using 'Tracked changes' in Word files as these are lost in PDF conversion.

I should be grateful if you would also provide a point-by-point response detailing how you have dealt with the points raised by the reviewers in the 'Response to Reviewers' box. Please attend to all of the reviewers' comments. If you do not agree with any of their criticisms or suggestions please explain clearly why this is so.

Reviewer 1

Advance summary and potential significance to field

The manuscript from Ji Ming Wang and colleges studies the role of the antimicrobial peptide CRAMP in the context of macrophages phagocytosis of E.coli. This manuscript demonstrates that CRAMP is expressed when macrophages are challenged with E.coli O22:H8. In addition, they demonstrated that CRAMP is required to inhibit intracellular bacterial replication. And finally, the involvement of autophagy in this process is elucidated. The current understanding of the role of CRAMP during E.coli infection is limited and this manuscript provides novel information on the function of CRAMP in macrophages. With minor corrections, I recommend this manuscript for publication in JCS.

Comments for the author

Comments from the first review were correctly addressed and changes have been included to improve this manuscript quality. A couple of small corrections that were required are still missing. The language has significantly improved.

© 2021. Published by The Company of Biologists under the terms of the Creative Commons Attribution License (https://creativecommons.org/licenses/by/4.0/).

There are only minor comments that should be adressed:
1.In the figures with different channels, it would be better to show the channel in grey and merge in colours. Or make DAPI appear in a different blue because when printed is difficult to visualize. And scale only in Merge.
2.It is mentioned that the de novo production of IkB-a in CRAMP+/+ compare to CREMP-/- at 2H and, but the quantification shows the opposite. Also, the phosphorylation of IkB-a is increased in CRAMP+/+, there is a chicken-egg situation between NfK-beta pathway activation and CRAMP expression where it is not clear who promotes in the first place the activation. This should be better to explain.
3.CRAMP in µg/ml should be corrected in the Fig. 2B title, to correspond with the quantification.
4.Fig. 2A In the x axe, better write Bay - + for each condition.
5.If CRAMP-/- is a knockout, why in the quantification of values are higher than zero?
6.In figure 4 “Inactivated E.coli is not needed” and X axes should be written like suggested for figure 2A.
7.In the supplementary Fig. 3 the quantification should be added to claim that p62 is higher in CRAMP+/+ compare to CRAMP-/-
8.A quantification of the co-localization between E.coli and LAMP-1 or p62 is required to claim that it is reduced or increased when comparing CRAMP+/+ or CRAMP-/-
9.In line 17 page 11, correct CRAMP-/- for CRAMP knockdown.
10.Always use MOI instead of saying the volume and concentration of bacteria used for infections. For instance, in the legends of the figures.
11.It is unnecessary to indicate the membrane of the cell with red arrows.

Reviewer 2

Advance summary and potential significance to field

The corrections made substantially improve the paper and I believe it is suitable for publication now.

Comments for the author

The corrections made substantially improve the paper and I believe it is suitable for publication now.

Reviewer 3

Advance summary and potential significance to field

The authors have adequately addressed all my questions and concerns.

Comments for the author

The authors have adequately addressed all my questions and concerns.

Second revision

Author response to reviewers' comments

Manuscript ID#: JOCES/2020/252148

Responses to the Reviewers’ comments and suggestions

© 2021. Published by The Company of Biologists under the terms of the Creative Commons Attribution License (https://creativecommons.org/licenses/by/4.0/).

We thank the Reviewer 2 and 3 for endorsement of the acceptance of our manuscript for publication by the Journal of Cell Science.

We also thank the Reviewer 1 for further and constructive comments and suggestions, which we address in the following point by point responses:

Reviewer 1
Q-1. In the figures with different channels, it would be better to show the channel in grey and merge in colors. Or make DAPI appear in a different blue because when printed is difficult to visualize. And scale only in Merge.

Answer: Thanks for the suggestion. We have removed scale bars except from "Merge" in Fig. 1B, Fig. 2A and Fig. 4.

Q-2. It is mentioned that the de novo production of IkB-a in CRAMP+/+ compare to CREMP-/- at 2H and, but the quantification shows the opposite. Also, the phosphorylation of IkB-a is increased in CRAMP+/+, there is a chicken-egg situation between NFκB-beta pathway activation and CRAMP expression where it is not clear who promotes in the first place the activation. This should be better to explain.

Answer: Thanks for the comment. We have verified the changes of IκB-α at 2 h. It is interesting to further investigate the question about 'chicken or egg".

Q-3. CRAMP in μg/ml should be corrected in the Fig. 2B title, to correspond with the quantification.

Answer: Thanks for the reminder. We have added the concentration for CRAMP in Fig. 2B (now Fig. 2D) of the revised manuscript.

Q-4. Fig. 2A In the x axe, better write Bay - + for each condition.

Answer: Thanks for the suggestion. We have modified the layout of Fig. 2A to make the Bay - + in the X axe.

Q-5. If CRAMP-/- is a knockout, why in the quantification of values are higher than zero?

Answer: Thanks for the question. We consider it as a rather non-specific background of staining by the antibody.

Q-6. In figure 4 "Inactivated E. coli is not needed" and X axes should be written like suggested for figure 2A.

Answer: Thanks for the suggestion. Fig. We have modified Fig. 4 as suggested.

Q-7. In the supplementary Fig. 3 the quantification should be added to claim that p62 is higher in CRAMP+/+ compare to CRAMP-/-

Answer: Thanks for the suggestion, we have quantified the level of p62 in Supplementary Fig. 3.

Q-8. A quantification of the co-localization between E. coli and LAMP-1 or p62 is required to claim that it is reduced or increased when comparing CRAMP+/+ or CRAMP-/-

Answer: The changes in the levels of LAMP-1 and p62 in macrophages are dynamic after stimulation with E. coli, depending on the activation of CRAMP in the cells. In this study, we focused on the relationship between CRAMP and activation of autophagy in macrophages. However, clarification of the relationship between E. coli infection and changes in autophagy elements is also an interesting and important issue for our future study.

Q-9. In line 17 page 11, correct CRAMP-/- for CRAMP knockdown.

Answer: We have corrected the error.

© 2021. Published by The Company of Biologists under the terms of the Creative Commons Attribution License (https://creativecommons.org/licenses/by/4.0/).

Q-10. Always use MOI instead of saying the volume and concentration of bacteria used for infections. For instance, in the legends of the figures.

Answer: We now have used MOI to replace the volume and concentration of bacteria in the legends to Fig. 1-5, Supplemental Fig. 2-3 as well as in Materials and methods.

Q-11. It is unnecessary to indicate the membrane of the cell with red arrows.

Answer: Thanks for the suggestion. We have removed red arrows in Fig. 3A.

We hope the further revised manuscript is now acceptable for publication in the Journal of Cell Science.

Third decision letter

MS ID#: JOCES/2020/252148

MS TITLE: Requirement of CRAMP for mouse macrophages to eliminate phagocytosed E. coli through an autophagy pathway

AUTHORS: Keqiang Chen, Teizo Yoshimura, Wanghua Gong, Cuimeng Tian, Jiaqiang Huang, and Ji-ming Wang
ARTICLE TYPE: Research Article

I am happy to tell you that your manuscript has been accepted for publication in Journal of Cell Science, pending standard ethics checks.

© 2021. Published by The Company of Biologists under the terms of the Creative Commons Attribution License (https://creativecommons.org/licenses/by/4.0/).
